# Preclinical development of carrier-free prodrug nanoparticles for enhanced antitumor therapeutic potential with less toxicity

**DOI:** 10.1186/s12951-022-01644-x

**Published:** 2022-10-04

**Authors:** Man Kyu Shim, Suah Yang, Jooho Park, Jun Sik Yoon, Jinseong Kim, Yujeong Moon, Nayeon Shim, Mihee Jo, Yongwhan Choi, Kwangmeyung Kim

**Affiliations:** 1 Medicinal Materials Research Center, Biomedical Research Institute, Korea Institute of Science and Technology, Seoul, 02792 Republic of Korea; 2 KU-KIST Graduate School of Converging Science and Technology, Korea University, Seoul, 02841 Republic of Korea; 3 Department of Bioengineering, Korea University, Seoul, 02841 Republic of Korea; 4 College of Pharmacy, Graduate School of Pharmaceutical Sciences, Ewha Womans University, Seoul, 03760 Republic of Korea

**Keywords:** Preclinical study, Prodrug, Nanoparticles, Targeted therapy, Cathepsin B, Doxorubicin

## Abstract

**Background:**

Nanomedicine has emerged as a promising strategy for cancer treatment. The most representative nanomedicine used in clinic is PEGylated liposomal doxorubicin DOXIL^®^, which is first FDA-approved nanomedicine. However, several shortcomings, such as low drug loading capacity, low tumor targeting, difficulty in mass production and potential toxicity of carrier materials, have hindered the successful clinical translation of nanomedicines. In this study, we report a preclinical development process of the carrier-free prodrug nanoparticles designed as an alternative formulation to overcome limitations of conventional nanomedicines in the terms of technical- and industrial-aspects.

**Results:**

The carrier-free prodrug nanoparticles (F68-FDOX) are prepared by self-assembly of cathepsin B-specific cleavable peptide (FRRG) and doxorubicin (DOX) conjugates without any additional carrier materials, and further stabilized with Pluronic F68, resulting in high drug loading (> 50%). The precise and concise structure allow mass production with easily controllable quality control (QC), and its lyophilized powder form has a great long-term storage stability at different temperatures (− 4, 37 and 60 °C). With high cathepsin B-specificity, F68-FDOX induce a potent cytotoxicity preferentially in cancer cells, whereas their cytotoxicity is greatly minimized in normal cells with innately low cathepsin B expression. In tumor models, F68-FDOX efficiently accumulates within tumor tissues owing to enhanced permeability and retention (EPR) effect and subsequently release toxic DOX molecules by cathepsin B-specific cleavage mechanism, showing a broad therapeutic spectrum with significant antitumor activity in three types of colon, breast and pancreatic cancers. Finally, the safety of F68-FDOX treatment is investigated after single-/multi-dosage into mice, showing greatly minimized DOX-related toxicity, compared to free DOX in normal mice.

**Conclusions:**

Collectively, these results provide potential preclinical development process of an alternative approach, new formulation of carrier-free prodrug nanoparticles, for clinical translation of nanomedicines.

**Supplementary Information:**

The online version contains supplementary material available at 10.1186/s12951-022-01644-x.

## Introduction

Among the Food and Drug Administration (FDA)-approved anticancer drugs, anthracyclines are the most widely applicable to treat various tumor types [1]. Doxorubicin (DOX), one of the most potent antineoplastic anthracyclines, is frequently used for chemotherapy in multiple solid tumors and hematological malignancies [2]. Commonly, DOX is used alone or in combination with other agents, remaining a central treatment option owing to its widest spectrum of activity [3, 4]. The antitumor efficacy of DOX is attributable to intercalate within the DNA helix and bind covalently to proteins that involve in DNA replication and transcription, resulting in ultimate cell death through inhibition of DNA, RNA and protein synthesis [5]. Despite its potent efficacy, the clinical use of DOX is strictly hindered owing to systemic toxicity accompanying severe cardiotoxicity by unfavorable pharmacokinetics and poor tumor targeting [6, 7]. Consequently, DOX-based chemotherapy generally demands for patients in good state who could tolerate the side effects; on the contrary, it is restricted the use in patients in serious and poor state who need chemotherapy [8].

Considerable efforts have been made to develop alternative strategies for reducing severe side effects of DOX [9, 10]. The most significant advances in clinic are the application of drug delivery systems using various nanomedicines [11, 12]. In particular, the first FDA-approved nanomedicine, DOXIL^®^, is a PEGylated liposomal DOX and based on three main principles: (i) liposome formulation with lipid bilayer in a “liquid ordered’ phase, composed of the high T_m_ (53 °C) of phosphatidylcholine and cholesterol; (ii) prolonged in vivo circulation time of drugs and avoidance of the reticuloendothelial system (RES) owing to the use of PEGylated liposomes; and (iii) fixable and stable remote drug loading by a transmembrane ammonium sulfate gradient methods, which allow drug-release at the tumors [13]. With these advantages, DOXIL^®^ can efficiently reduce the side effects of DOX owing to tumor-targeted delivery by enhanced permeability and retention (EPR) effect [14]. The successful “first in man” clinical trials of DOXIL^®^ with overall patient survival improvement prompted human use of first generation of nanomedicine, and it was approved from FDA in 1995 [13].

However, additional approval for clinical use of nanomedicines, including liposomes as well as polymeric nanoparticles, dendrimers, micelles, inorganic nanoparticles have failed because of their several shortcomings [15, 16]. First, these carrier materials have low drug loading contents (< 10%) and the risks of potential toxicity and immunogenicity [17]. In addition, their structures and synthetic processes are fairly complex, hindering precise quality control (QC) and scale-up industrial production [18, 19]. Notably, recent studies have noted unexpectedly low delivery efficiency of nano-sized drug delivery system, with less than 1% of the administered nanomedicines being targeted to the tumors in many preclinical models [20]. Therefore, 99% of nanomedicine exist in off-target tissues, leading to severe side effects by carrier material-induced toxicities and non-specific drug leakage [21]. As a result, considerable amounts of drugs are non-specifically distributed in normal tissues and blood, which induce severe systemic toxicity.

We have recently proposed new formulation of carrier-free prodrug nanoparticles for DOX delivery to enhance antitumor therapeutic potential with less toxicity in normal tissues [22]. The new carrier-free prodrug nanoparticles are prepared by self-assembly of cancer-specific prodrugs, constructed with tumor-overexpressed cathepsin B-specific cleavable peptide FRRG and DOX (FRRG-DOX). The direct conjugation of FRRG peptide and DOX is enable to avoid premature drug leakage in off-target tissues; furthermore, their precise and concise structure is easy to achieve mass production with controllable QC. In particular, FRRG-DOX molecules spontaneously self-assembled into prodrug nanoparticles via intermolecular hydrophobic interactions without any additional carrier materials, thereby allowing novel nanoparticle system consisting with only drug molecules [23–25]. These carrier-free nanoparticles can overcome the fundamental problem of conventional nanoparticle system showing low drug loading capacity with less than 10%. Therefore, many carrier-free nanoparticles have been developed with considerably high drug loading contents (DLC) of various anticancer agents, such as cabazitaxel (86%) [26, 27], cabazitaxel and chlorin e6 (DLC: 64.65%) [28], cabazitaxel and dasatinib (100%) [29], dasatinib (100%) [30], and mycophenolate mofetil (72.9%) [31]. Consequently, FRRG-DOX nanoparticles induce a significant cytotoxicity in cathepsin B-overexpressed tumor tissues by releasing toxic DOX molecules, while DOX release is mitigated in cathepsin B-deficient normal tissues to minimize DOX-related side effects. In our first study, the safe and effective chemotherapy by FRRG-DOX nanoparticles was demonstrated in preclinical colon tumor models [22]. As a following study, we further stabilized the prodrug nanoparticles with the FDA-approved excipient, Pluronic F68, to increase the stability of particle structure and used it for combination with anti-PD-L1 antibody for cancer immunotherapy [32]. The FRRG-DOX nanoparticles induced preferential immunogenic cell death (ICD) in tumor cells with minimal toxicity towards immune cells, resulting in potent immune checkpoint blockade therapy compared to free DOX when combined with PD-L1 antibody. Finally, we were also interested in testing FRRG-DOX nanoparticles for intraperitoneal (I.P.) drug delivery in a peritoneal metastatic ovarian carcinomatosis models [33]. When intraperitoneally injected, they efficiently prolonged in vivo residence time by reducing rapid absorption to normal tissues and targeted the peritoneal carcinomatosis via the two different targeting mechanisms of direct penetration and systemic blood vessel-associated accumulation, which greatly improved the therapeutic potential of DOX with less toxicity.

**Scheme 1.**
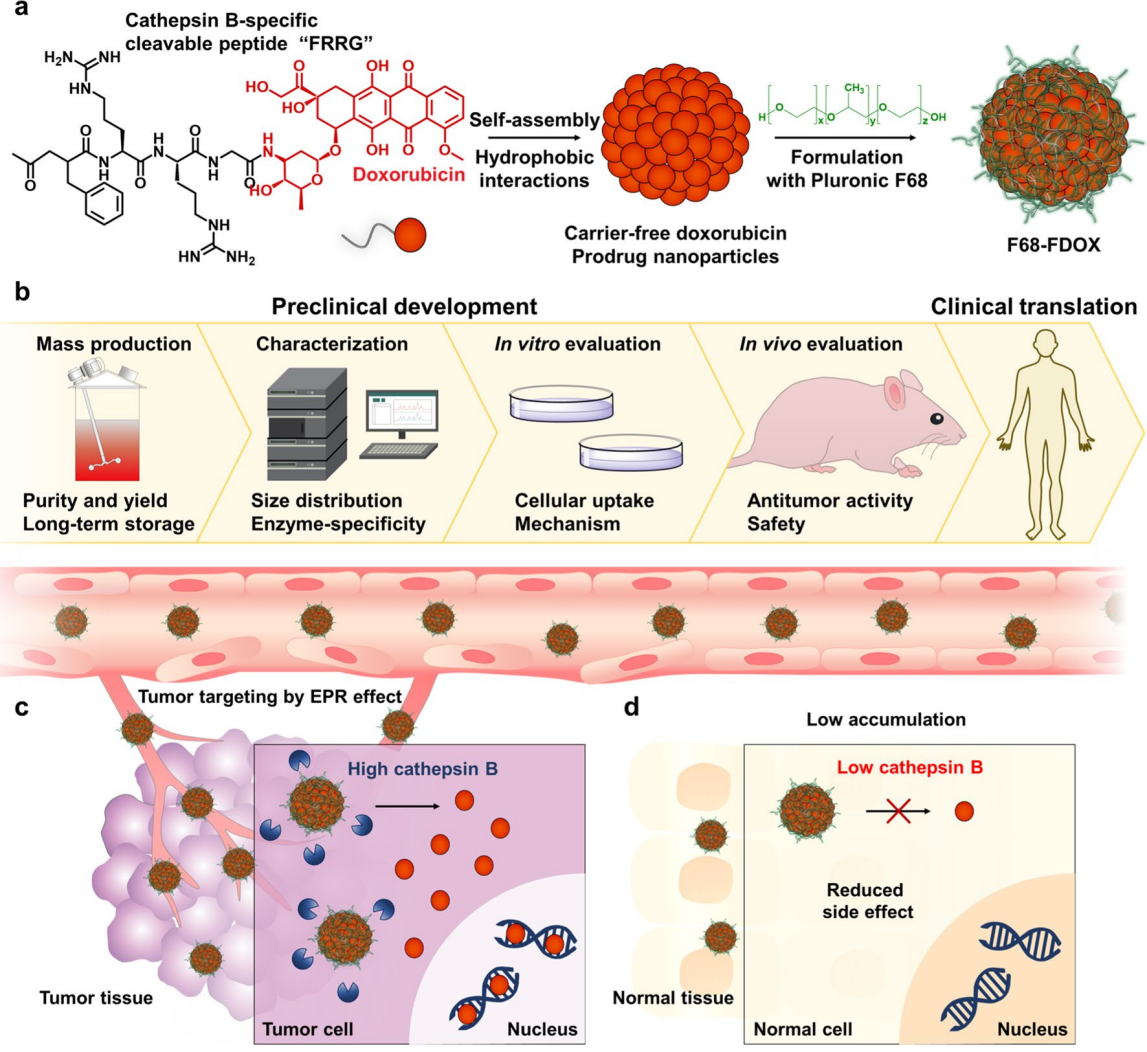
Preclinical development of carrier-free prodrug nanoparticles (F68-FDOX) for enhanced antitumor therapeutic potential with less toxicity. **a** The cancer-specific prodrug was simply prepared by conjugating cathepsin B-specific cleavable tetrapeptide (Phe-Arg-Arg-Gly; FRRG) to doxorubicin (DOX). The resulting FRRG-DOX spontaneously self-assembled into nanoparticles via intermolecular hydrophobic interactions without any additional carrier materials and further stabilized with FDA-approved excipient, Pluronic F68. **b** The preclinical development process of F68-FDOX, such as mass production, characterization, and in vitro and in vivo evaluation is studied. **c** F68-FDOX efficiently accumulates within tumor tissues by enhanced permeability and retention (EPR) effect and subsequently release the DOX by cathepsin B-specific cleavage mechanism, showing a potent antitumor activity in colon, breast and pancreatic cancers. **d** The F68-FDOX greatly minimize the DOX-related systemic toxicity by maintaining inactive state in normal tissues with innately low cathepsin B

These successful investigations have motivated the preclinical development of carrier-free DOX prodrug nanoparticles, which is now underway. On the practical aspect, establishing industrial-scale manufacturing of nanomedicine is a most important task. Even if nanomedicines can be prepared on a small-scale for academic use, industrial-scale manufacturing can still be controversial in terms of function and quality. In present study, we optimize a manufacturing operation for mass production of FRRG-DOX stabilized with Pluronic F68 **(**F68-FDOX; Scheme 1a). For preclinical study, we develop F68-FDOX as a lyophilized powder form and assess long-term storage stability, characterization, in vitro cellular uptake mechanism, and, in vivo antitumor activity and safety (Scheme 1b). The PK/PD profiles of F68-FDOX and their tumor targeting by EPR effect are assessed in preclinical colon tumor models (Scheme 1c). Furthermore, the antitumor activity is investigated in three types of preclinical models with refractory tumors. Finally, safety of F68-FDOX treatment is evaluated in vivo after single- or multi-dosage (Scheme 1d). These series of studies focused on industrial consideration for clinical use of nanomedicine, such as optimizing process for scalable synthesis, testing long-term stability in the various condition to assure quality of the samples, establishing how to use for in vivo administration, and screening ultimate target tumor types that can expect superior antitumor efficacy. Thus, this study can provide a preclinical development process for clinical translation of new nanomedicine for targeted cancer treatment.

## Results and discussion

### Preparation of carrier-free prodrug nanoparticles for preclinical development

**Fig. 1:**
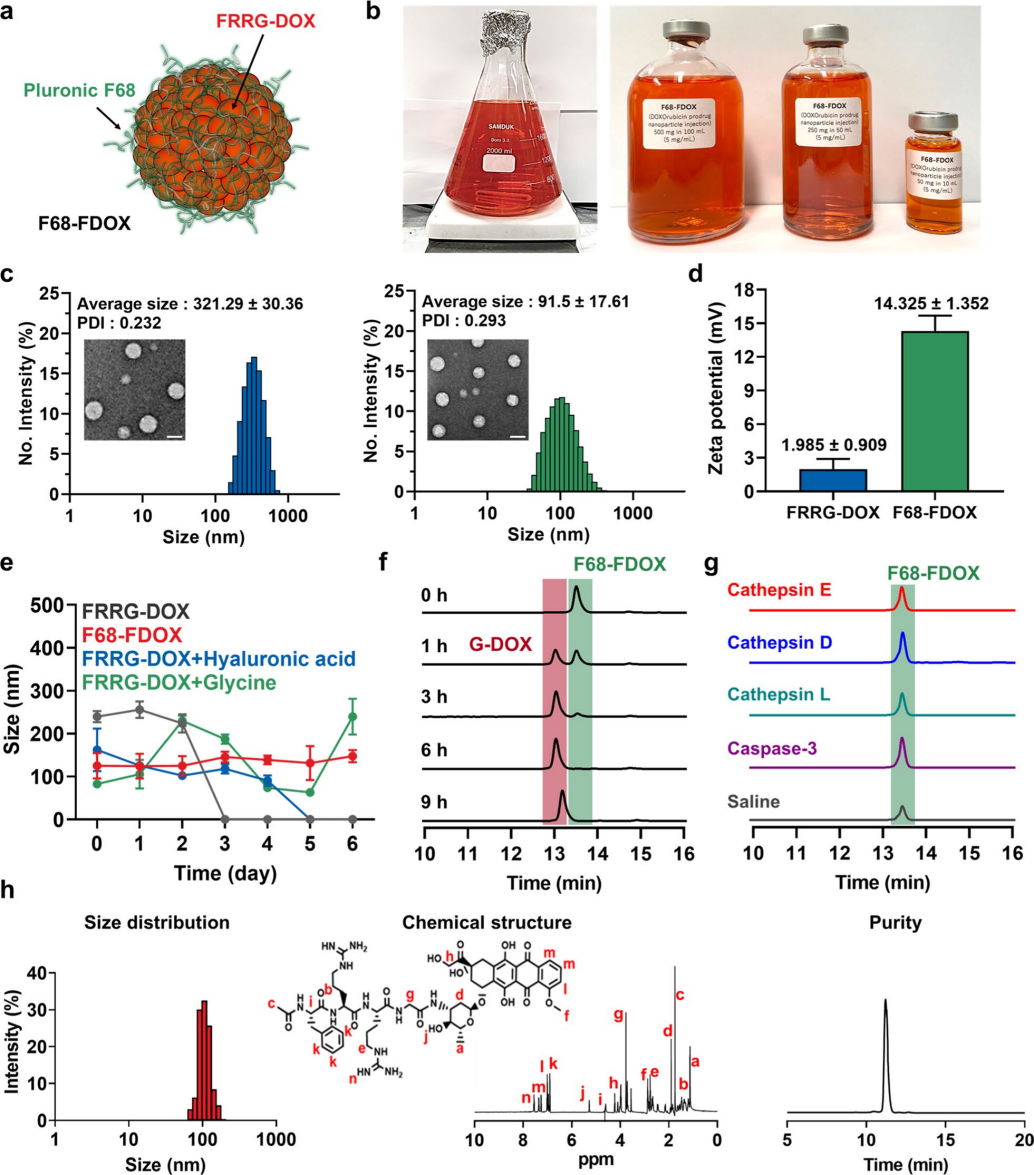
Preparation of carrier-free prodrug nanoparticles (F68-FDOX) for preclinical development. **a** Schematic illustration showing structure of F68-FDOX. **b** Picture to show large scale synthesis of F68-FDOX at one batch up to 200 g in 2 L volume. **c** Size distribution and morphology of FRRG-DOX and F68-FDOX nanoparticles. **d** Zeta potential of FRRG-DOX and F68-FDOX nanoparticles. **e** Size stability of FRRG-DOX and FRRG-DOX stabilized with Pluronic F68 (F68-FDOX), hyaluronic acid or glycine nanoparticles in mouse serum. **f–g** Cleavage behavior of F68-FDOX after incubation with **f** cathepsin B or **g** other enzymes (cathepsin E, D, L and caspase-3) or saline (hydrolysis). **h** The size distribution, chemical structure and purity after 24 h reconstitution of lyophilized F68-FDOX powder stored at low (− 4 °C) temperature for 12 months

The carrier-free doxorubicin (DOX) prodrug nanoparticles were designed as an alternative formulation to overcome several problems of conventional nano-sized drug delivery system in the terms of technical- and industrial-aspects. First, the cancer-specific prodrug was simply prepared by conjugating cathepsin B-specific cleavable tetrapeptide (Phe-Arg-Arg-Gly; FRRG) to DOX via one-step reaction (Additional file 1: Fig. S1). This absolutely simplified one-step synthesis protocol allowed 100 g batch of preparation as described in “Methods” section. FRRG peptide have high-specificity towards the target bioenzyme of cathepsin B to trigger drug release from prodrug in the targeted tumor cells and maintain non-toxic inactive state in normal cells with innately low cathepsin B expression, leading to enhanced antitumor therapeutic potential with less toxicity [22, 25, 34, 35]. In addition, their precise and concise structure allow easy quality control (QC) after synthesis; thus, we could verify the successful preparation of FRRG-DOX by confirming chemical structure, exact mass and purity via ^1^H NMR, MALDI-TOF (calculated mass: 1102.17 Da, measured mass: 1102.595 m/z) and HPLC (99%), respectively (Additional file 1: Fig. S2). Importantly, FRRG-DOX molecules self-assembled into prodrug nanoparticles by its intermolecular π-π stacking hydrophobic interactions without any additional carrier materials, resulting in high drug loading (> 50%) [23, 24]. To enhance the in vivo stability, FRRG-DOX nanoparticles were further stabilized with clinically validated pharmaceutical excipient, Pluronic F68 (30% w/w) through simple drop casting method, resulting in F68-FDOX (Fig. 1a). The conventional nano-formulations are normally prepared by resolving raw material medicine and excipient in the solvent with good solubility, followed by lyophilization and re-dispersed in the aqueous condition [36]. However, F68-FDOX was prepared in the aqueous condition that FRRG-DOX molecules exist as a prodrug nanoparticle, for stabilization by surface coating with Pluronic F68. The resulting F68-FDOX was prepared by adding FRRG-DOX solution to the Pluronic F68 solution under the distilled water condition; this simple procedure allowed us to accomplish large scale batch up to 200 g in 2 L volume (Fig. 1b). The F68-FDOX in aqueous condition showed spherical structure with average size of 91.5 ± 17.61 nm, which became smaller after surface coating of FRRG-DOX nanoparticles (321.29 ± 30.36 nm) with Pluronic F68 (Fig. 1c). This is attributable to the formulation with nonionic emulsifier Pluronic F68 that provides an additional steric stabilization effect to prevent aggregation of fine particles, resulting in narrow size distribution and smaller particle size [37]. In addition, the zeta potential of F68-FDOX was also significantly increased than FRRG-DOX owing to the presence of positively charged Pluronic F68 layer on the particle surface (Fig. 1d). As a result, FRRG-DOX nanoparticles were dissociated in mouse serum within 3 days of incubation, while F68-FDOX showed high stability without significant changes of the size and polydispersity index for 6 days (Fig. 1e and Additional file 1: Fig. S3). Importantly, the particle structure of F68-FDOX in the mouse serum was also highly stable in comparison to the FRRG-DOX stabilized with hyaluronic acid or glycine (30% w/w), indicating great suitability of Pluronic F68 as a pharmaceutical excipient to improve the stability of FRRG-DOX. These stable characteristics of F68-FDOX in the physiological condition is suitable to accumulate within tumor tissues via EPR effect in vivo [14]. Next, cathepsin B-specific cleavage of F68-FDOX was confirmed in various conditions. When the F68-FDOX was incubated with MES buffer (pH 5.5) including cathepsin B at 37 °C, 99.53% of F68-FDOX was cleaved to glycine-conjugated DOX (G-DOX) within 9 h post-incubation (Fig. 1f and Additional file 1: Fig. S4); the enzymatic cleavage after incubation of F68-FDOX with cathepsin B was slightly delayed compared to FRRG-DOX owing to reduced enzyme accessibility by surface coating with Pluronic F68 (Additional file 1: Fig. S5). This was clearly supported by MALDI-TOF measurement, wherein the molecular weights of G-DOX (calculated mass: 600.58 Da, measured mass: 656.4 m/z [M + Li] and 657.4 m/z [M + Li + H]) were confirmed at the newly appeared peak (13 min) in the HPLC spectrum after incubation of F68-FDOX with cathepsin B (Additional file 1: Fig. S6). It was already reported that one or two glycine (G) or leucine (L) peptide sequences are cleaved by intracellular lysosomal proteases when they are chemically conjugated to the DOX molecules [38]. Furthermore, previous studies showed that G-DOX cleaved from FRRG-DOX efficiently metabolized into free DOX in cultured cancer cells [21, 32]. In contrast, F68-FDOX was not cleaved when incubated with cathepsin E, D, L or caspase-3 for 24 h (Fig. 1g).

Finally, we developed F68-FDOX as a lyophilized powder form and evaluated the long-term storage stability of lyophilized F68-FDOX powder stored for 3, 6, 12 months in the low (− 4 °C), room (37 °C) or accelerated (60 °C) condition; for these studies, size distribution, chemical structure and purity were analyzed after reconstitution of lyophilized powder stored at each condition (Additional file 1: Fig. S7–S9). The results showed homogeneous size distribution without chemical degradation and impurity formation similar to those of freshly prepared F68-FDOX, in all different conditions, indicating excellent storage stability of lyophilized power form. We also performed same experiment after 24 h of reconstitution using lyophilized F68-FDOX power stored at 12 months in the low temperature, which are considered as a similar condition with clinical use of DOXIL^®^; no significant changes were observed in size distribution, chemical structure and purity (Fig. 1h). Taken together, the manufacturing operation for mass production of F68-FDOX was optimized for preclinical development, and their physicochemical characterization, such as size distribution, particle stability, target enzyme-specificity, and even the long-term storage stability was successfully evaluated in vitro.

### Cellular uptake and cancer cell-specific cytotoxicity of F68-FDOX

**Fig. 2:**
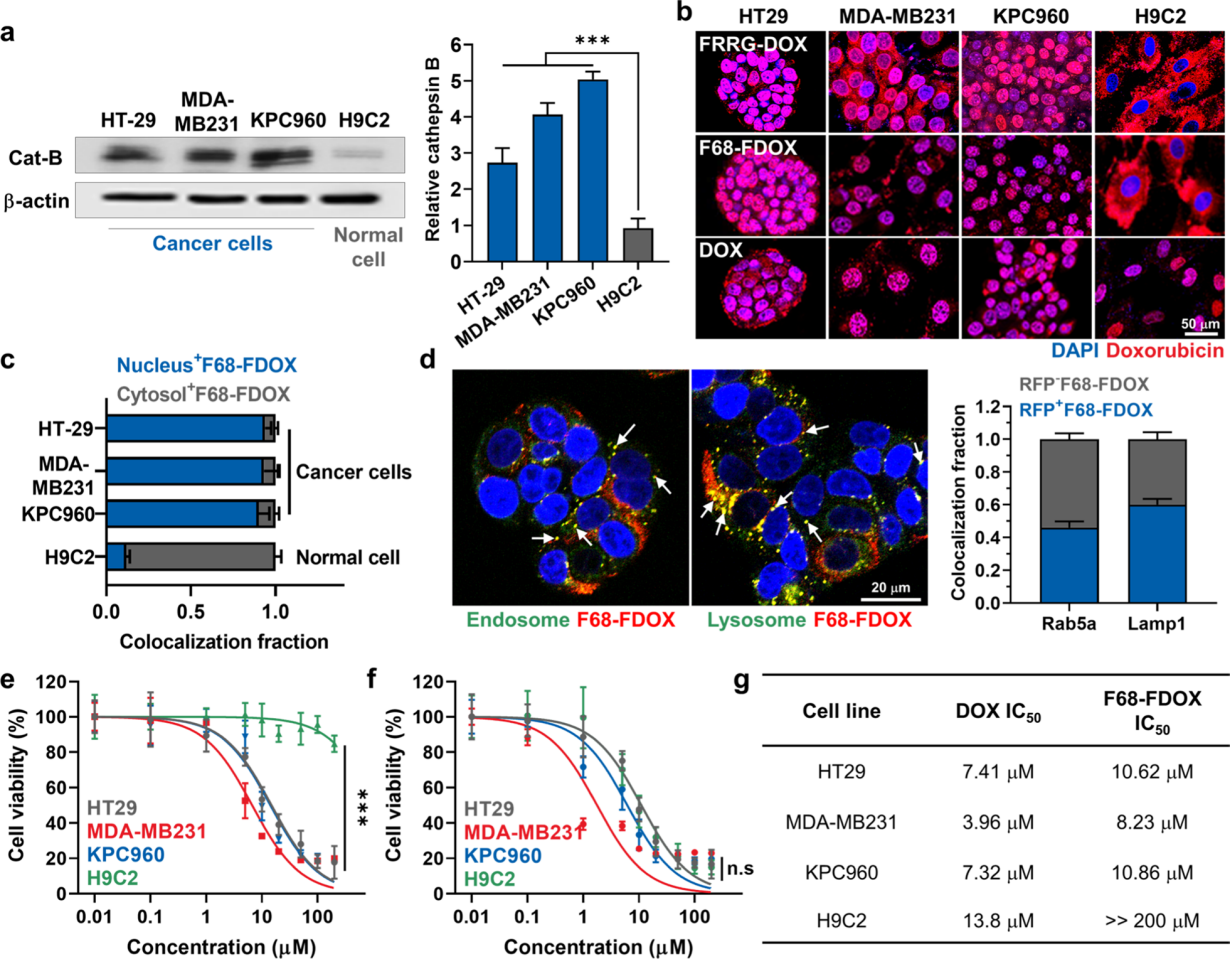
Cellular uptake and cancer cell-specific cytotoxicity of F68-FDOX. **a** The cathepsin B expression levels in the HT29, MDA-MB231, KPC960 and H9C2 cells. **b** Cellular uptake of FRRG-DOX, F68-FDOX and DOX in the HT29, MDA-MB231, KPC960 and H9C2 cells after 48 h of incubation. **c** Quantitative analysis for the amount of F68-FDOX in the cytosol or nucleus in different cells after 48 h of incubation. **d** Fluorescence images of HT29 cells labeled with Rab5a-RFP (endosomes) or Lamp1-RFP (lysosomes) after 48 h of F68-FDOX treatment. **e**,** f** The cell viability of HT29, MDA-MB231, KPC960 and H9C2 cells after 48 h treatment with **e** F68-FDOX or **f** DOX. **g** The IC50 values of F68-FDOX and DOX in the HT29, MDA-MB231, KPC960 and H9C2 cells. Significance was determined by Tukey − Kramer *post-hoc* test

The cellular uptake of F68-FDOX was assessed in three types of cancer cells (HT29, human colon adenocarcinoma; MDA-MB231, human breast adenocarcinoma; KPC960, human pancreatic ductal adenocarcinoma) and normal cell (H9C2, rat cardiomyocytes). As expected, three types of cancer cells expressed a 4.78–8.04-fold higher amount of cathepsin B than H9C2 cells (Fig. 2a) [39]. The F68-FDOX and FRRG-DOX showed robust cellular uptake in a time-dependent manner in all types of cells (Fig. 2b and Additional file 1: Fig. S10). Importantly, a strong DOX fluorescence signals (red color) were observed limited to the nuclei of three types of cancer cells owing to internalization of DOX molecules into the nuclei after rapid cleavage by cathepsin B (Additional file 1: Fig. S11). In addition, molecular weight of free DOX in all cancer cells treated with F68-FDOX for 48 h was clearly detected by MALDI-TOF (calculated mass: 543.53 Da, measured mass: 568.2 m/z [M + Na + H]), indicating successful metabolism of G-DOX cleaved from F68-FDOX into free DOX (Additional file 1: Fig. S12). In contrast, F68-FDOX was mainly observed in the perinuclear compartment and cytosol of the cathepsin B-deficient H9C2 cells. Quantitatively, the DOX fluorescence signals in nuclei of F68-FDOX-treated cancer cells (HT29, MDA-MB231 and KPC960) were 7.7–8.0-fold stronger than H9C2 normal cells treated with F68-FDOX after 48 h of incubation (Fig. 2c). Since DOX induces a potent cytotoxicity by DNA intercalation in the nucleus, these intracellular behaviors of F68-FDOX can lead to the cancer-cell specific cytotoxicity, which minimize side effects toward off-target tissues by cathepsin B-specific cleavage mechanism. Next, cellular uptake mechanism of F68-FDOX was assessed in HT29 cells, which express Rab5a–RFP (a marker for early endosomes) or Lamp1–RFP (a marker for lysosomes), respectively. When the HT29 cells were incubated with F68-FDOX (1 μM) for 6 h at 37 °C, approximately 40% of F68-FDOX was observed in the endosomes, and that of 60% localized in the lysosomes (F68-FDOX observed in the endosomes or lysosomes were marked with white arrows, Fig. 2d). These results indicate that F68-FRRG-DOX internalize into the cells through endosomal/lysosomal pathway. Since a lysosomal protease, cathepsin B exhibits the highest enzymatic activity in acidic environment (pH 4–5) of lysosomes, this endocytosis route of F68-FDOX is suitable to enhance cathepsin B-specific drug release [40]. In agreement with the above in vitro results, the IC_50_ values of F68-FDOX were measured to be 10.62, 8.23 and 10.86 μM in HT29, MDA-MB231 and KPC960 after 48 of incubation, respectively (Fig. 2e); as a control, the cytotoxicity of FRRG-DOX in each cell was similar with F68-FDOX (Additional file 1: Fig. S13). In contrast, F68-FDOX exhibited > 200 μM of IC_50_ value in H9C2 cells, showing about a 20-fold difference between cancer and normal cells. As a control, DOX induced indiscriminate cytotoxicity with similar IC_50_ values in all cancer and normal cells (Fig. 2f and g). These results clearly demonstrate that F68-FDOX induce cytotoxicity preferentially in the cancer cells by cathepsin B-specific cleavage after endosomal/lysosomal uptake, while maintain inactive state in cathepsin B-deficient normal cells.

### PK/PD and tumor targeting of F68-FDOX

**Fig. 3:**
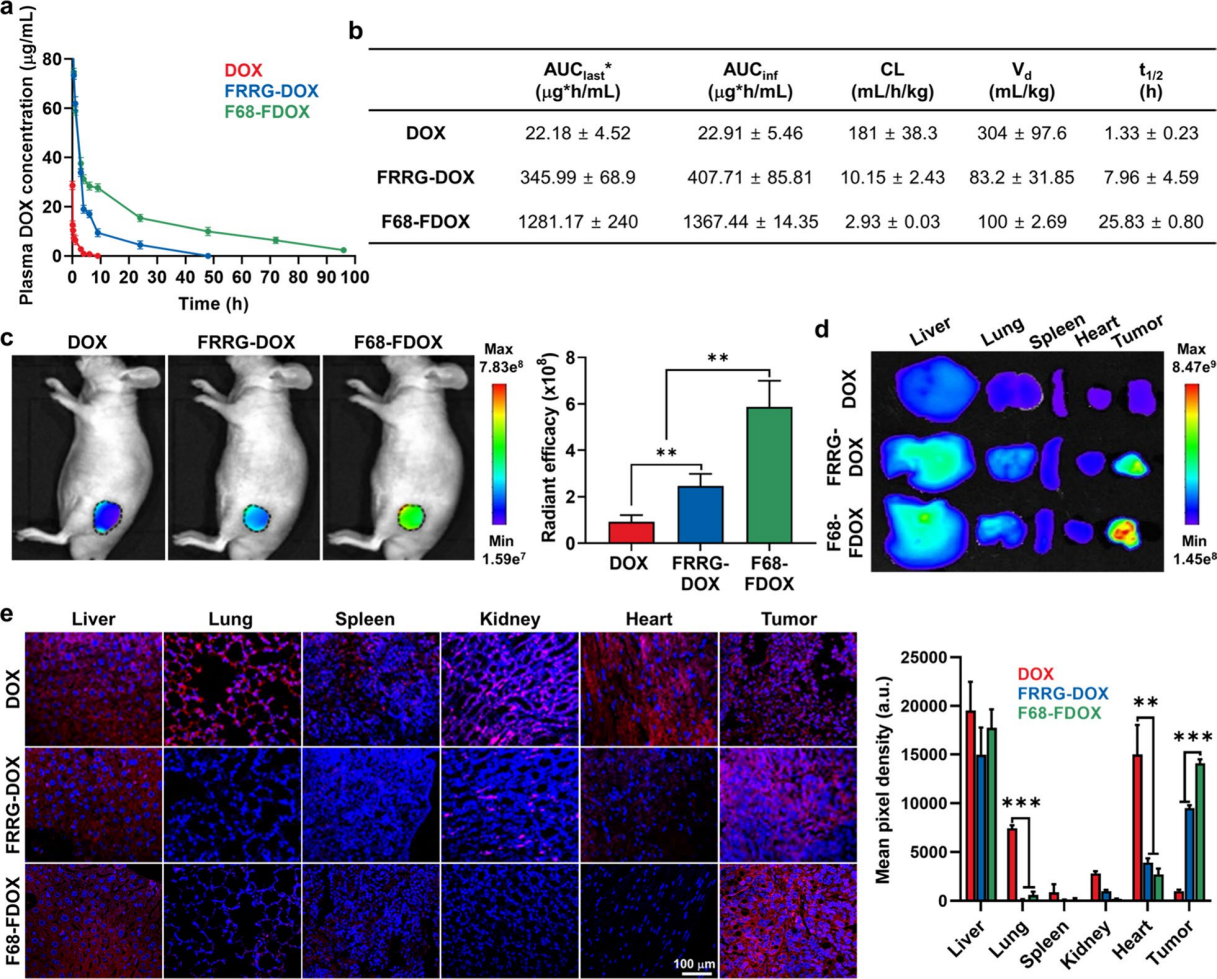
PK/PD and tumor targeting of F68-FDOX. **a** The pharmacokinetics (PK) profile of DOX, FRRG-DOX and F68-FDOX in mice. **b** The area under the curves (AUC), clearance (CL), volume of distribution (Vd) and half-life (*t*_1/2_) of DOX, FRRG-DOX and F68-FDOX in mice. **c** NIRF images of HT29 tumor-bearing mice after 9 h treatment with DOX, FRRG-DOX and F68-FDOX. **d** The ex vivo imaging of major organs and tumor tissues of HT29 tumor-bearing mice after 9 h treatment with DOX, FRRG-DOX and F68-FDOX. **e** The pharmacodynamics (PD) of DOX, FRRG-DOX and F68-FDOX in HT29 tumor-bearing mice after 9 h treatment**.** Significance was determined by Tukey−Kramer *post-hoc* test

To evaluate enhanced biodistribution and tumor targeting of F68-FDOX, their pharmacokinetics (PK) profile was compared to DOX and FRRG-DOX in BALB/c nu/nu mice. For this analysis, equivalent 4 mg/kg dose based on DOX contents of free DOX, FRRG-DOX or F68-FDOX were intravenously injected into the mice, and blood samples were collected at pre-determined times. Interestingly, DOX showed fast in vivo clearance with a short half-life (*t*_1/2_) of 1.33 ± 0.23 h, whereas FRRG-DOX exhibited a significantly extended *t*_1/2_ of 7.96 ± 4.59 h **(**Fig. 3a). Notably, F68-FDOX showed greatly prolonged *t*_1/2_ of 25.83 ± 0.8 h, which is attributable to the steric stabilization effect by stabilization with Pluronic F68. In addition, a detectable amount of the F68-FDOX remained for 96 h in the body, showing the dramatically extended residence time in vivo. The various PK parameters, such as area under the curves (AUC), clearance (CL) and volume of distribution (Vd) of F68-FDOX were also greatly improved compared to those of DOX and FRRG-DOX, thereby further confirming longer blood plasma half-life (Fig. 3b). Motivated by the greatly improved PK profiles of F68-FDOX, we assessed tumor targeting in the HT29 tumor-bearing mice, which were prepared by subcutaneous inoculation of 1 × 10^7^ of HT29 cells. When the tumor volumes were approximately 200 mm^3^, free DOX (4 mg/kg), FRRG-DOX (4 mg/kg based on DOX contents) or F68-FDOX (4 mg/kg based on DOX contents) were intravenously injected into the mice, followed by noninvasive near-infrared fluorescence imaging (NIRF). The NIRF images showed the significantly high tumor accumulation of F68-FDOX after 9 h of injection, wherein the fluorescence intensity of F68-FDOX in the tumor tissues was 6.33–6.82-fold and 2.42–2.71-fold stronger than DOX and FRRG-DOX, respectively (Fig. 3c). In addition, the ex vivo fluorescence imaging of major organs and tumor tissues after 9 h of injection further confirmed the enhanced tumor targeting of F68-FDOX (Fig. 3d and Additional file 1: Fig. S14). The histological analysis of major organs and tumor tissues was further performed after 9 h of injection for confirming more reliable pharmacodynamics (PD) of F68-FDOX; this is because the NIRF intensity of DOX is not large enough in vivo to precisely assess the biodistribution. The results exhibited that DOX was non-specifically distributed in all the major organs and low tumor accumulation, whereas FRRG-DOX highly accumulated in the tumor tissues with less distribution in the off-target tissues (Fig. 3e). Most importantly, F68-FDOX showed most high tumor accumulation owing to the favorable PK with prolonged in vivo residence time for EPR effect, wherein the 14.12–15.01-fold and 1.5–1.580-fold higher DOX fluorescence was observed in the tumor tissues of mice treated with F68-FDOX compared to that of DOX and FRRG-DOX, respectively. Taken together, F68-FDOX efficiently improve the PK/PD profiles of DOX, which significantly enhance the tumor accumulation and mitigate the distribution in the off-target tissues.

### In vivo antitumor activity of F68-FRRG-DOX

**Fig. 4:**
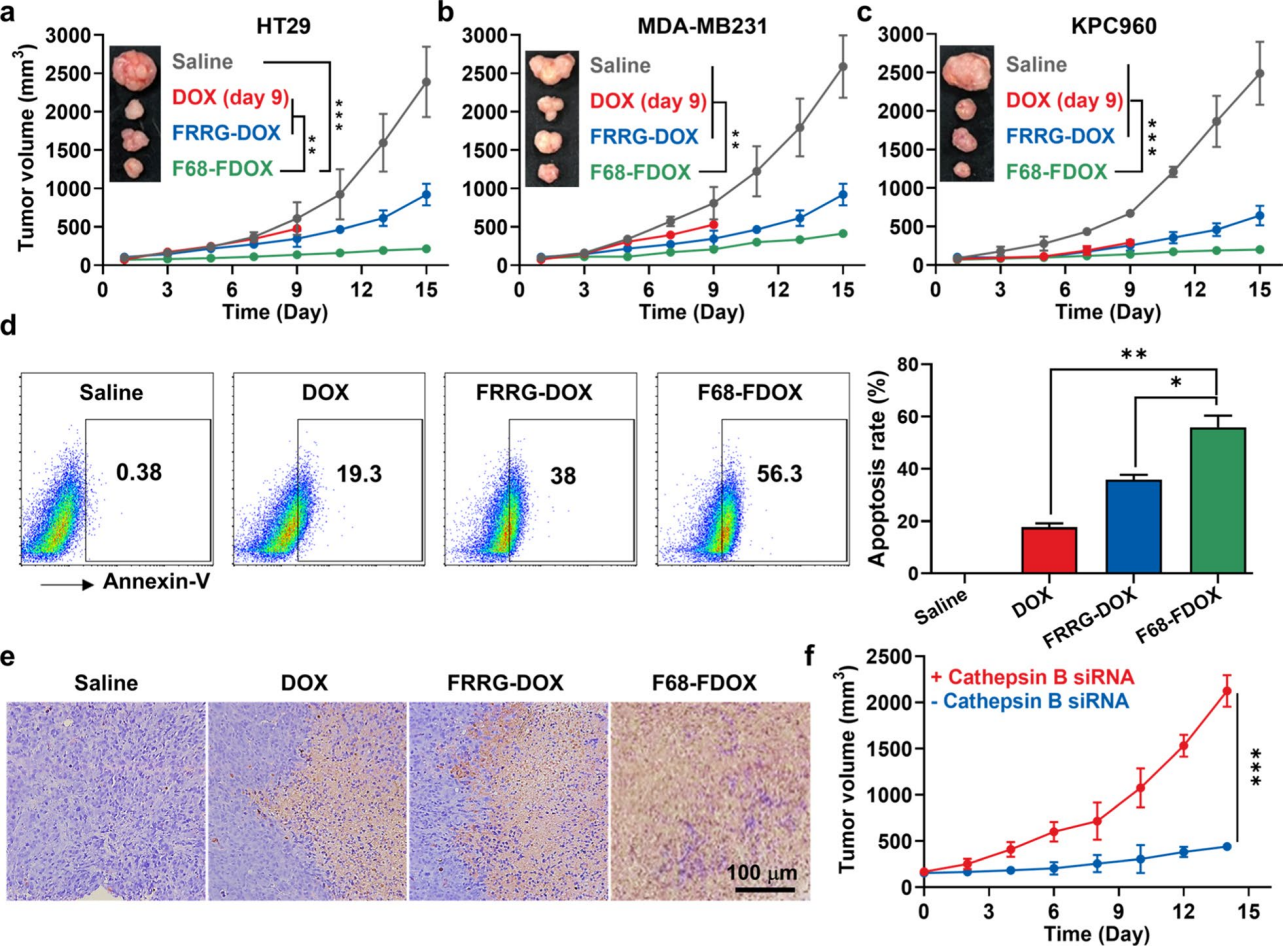
In vivo antitumor activity of F68-FRRG-DOX. **a–c** Tumor growth of **a** colon (HT29) tumor-, **b** breast (MDA-MB231) tumor- and **c** pancreatic (KPC960) tumor-bearing mice during treatment with DOX, FRRG-DOX or F68-FDOX once every three days. **d** The percentage of Annexin V-positive tumor cells after 9 days of treatment. **e** Tumor tissues stained with TUNEL on day 9 after treatment. **f** Tumor growth of mice treated with F68-FDOX along with cathepsin B-inhibitory siRNA or alone. Significance was determined by Tukey−Kramer *post-hoc* test (**a**–**d**) or Student's *t* test (**f**)

The antitumor activity of F68-FDOX was assessed in the mice models bearing three types of refractory tumors because one of the key challenges commonly encountered in drug discovery is that antitumor therapeutic potential evaluated with one tumor models do not necessarily translate across different tumor models [41]. The colon, breast and pancreatic tumor models were prepared by subcutaneous inoculation of 1 × 10^7^ of HT29, MDA-MB231 or KPC960, respectively; then, DOX (4 mg/kg), FRRG-DOX (4 mg/kg based on DOX) or F68-FDOX (4 mg/kg based on DOX) were intravenously injected once every three days when the tumor volumes were approximately 80 mm^3^. As expected, F68-FDOX (137.67 ± 21.61 mm^3^) significantly delayed the colon tumor growth compared to saline (608.65 ± 210.67 mm^3^, P < 0.001), DOX (478.75 ± 49.87 mm^3^, P < 0.01) and FRRG-DOX (347.29 ± 107.48 mm^3^, P < 0.01) on day 9 after treatment (Fig. 4a). In case of DOX-treated group, all the mice were dead within 9 days owing to the severe systemic toxicity. In addition, the potential antitumor activity of F68-FDOX was also observed in the breast and pancreatic tumor models, showing significantly inhibited tumor progression compared to saline (breast tumor, P < 0.01; pancreatic tumor, P < 0.001), DOX (breast tumor, P < 0.01; pancreatic tumor, P < 0.001) and FRRG-DOX **(**breast tumor, P < 0.01; pancreatic tumor, P < 0.001; Fig. 4b and c). These results demonstrate the broad therapeutic spectrum of F68-FDOX for the refractory tumors in clinic. The Annexin V staining of single tumor cells from colon tumor tissues further confirmed enhanced antitumor activity of F68-FDOX on day 9 after treatment, wherein the percentage of apoptotic cells was significantly higher in the F68-FDOX group (55.93 ± 4.46%) than in saline (0.4 ± 0.02%), DOX (17.7 ± 1.51%) and FRRG-DOX (35.87 ± 1.87%) groups (Fig. 4d). Tumor tissues stained with TUNEL also showed greatly elevated apoptosis region in tumor tissues of mice treated with F68-FDOX compared to saline (P < 0.001), DOX (P < 0.01) and FRRG-DOX **(**P < 0.05; Fig. 4e and Additional file 1: Fig. S15). Finally, we examined the in vivo cathepsin B-specificity of F68-FDOX with two groups of colon tumor models: (i) F68-FDOX treatment once every three days along with the local injection with the cathepsin B-inhibitory siRNA 7 times with 2 days-intervals; and (ii) F68-FDOX treatment under the same protocol. Interestingly, co-treatment with cathepsin B-inhibitory siRNA significantly inhibited the antitumor activity of F68-FDOX; as a result, the volumes of tumors (2123.87 ± 171.56 mm^3^) rapidly increased compared to those of mice treated with F68-FDOX only (438.26 ± 22.55 mm^3^), on day 15 after treatment (Fig. 4f). These results clearly indicate that F68-FDOX have a broad spectrum of antitumor activity against refractory tumor types and their high in vivo cathepsin B-specificity can be expected to mitigate the DOX-related side effects by maintaining inactive state in cathepsin B-deficient normal tissues.

### Safety of F68-FDOX treatment

**Fig. 5:**
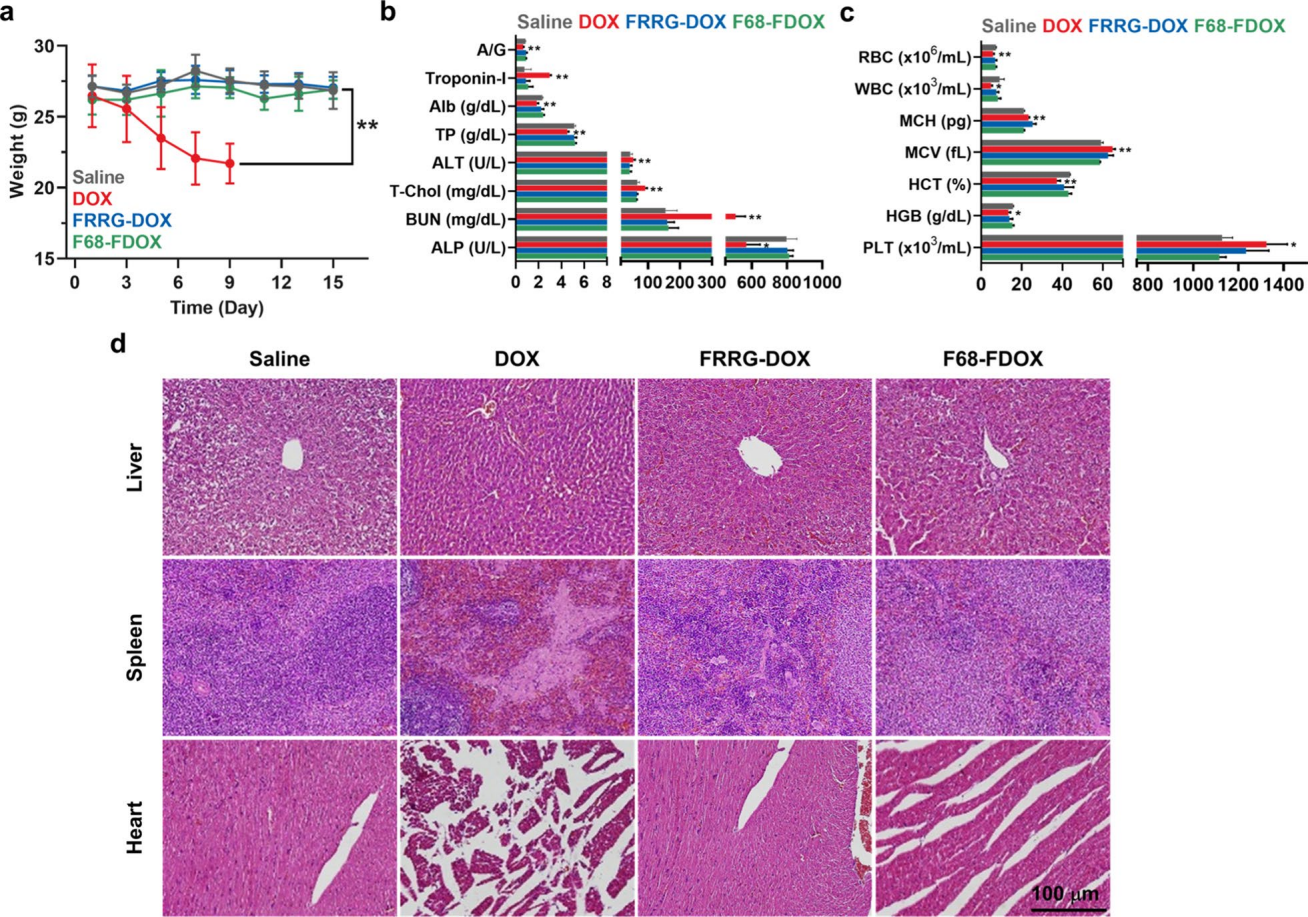
Toxicity study after single-dosage. **a** Body weight change after single-dosage with DOX, FRRG-DOX or F68-FDOX. **b** The serological examination on day 9 after single-dosage with DOX, FRRG-DOX or F68-FDOX. **c** The complete blood count **(**CBC) analyses on day 9 after single-dosage with DOX, FRRG-DOX or F68-FDOX. **d)** Major organ tissues stained with H&E on day 9 after single-dosage with DOX, FRRG-DOX or F68-FDOX. Significance was determined by Tukey−Kramer *post-hoc* test

The safety of F68-FDOX treatment was evaluated in the BALB/c mice after single-/multi-dosage. The DOX (10 mg/kg), FRRG-DOX (10 mg/kg based on DOX) or F68-FDOX (10 mg/kg based on DOX) were intravenously injected into the mice. First, body weight of the mice treated with DOX gradually reduced after treatment due to their severe systemic toxicity (Fig. 5a). In contrast, F68-FDOX- and FRRG-DOX-treated mice showed no significant body weight loss compared to saline-treated group. Consequently, mice in the DOX group were all dead within 9 days of treatment, whereas F68-FDOX-treated mice were survived for up to 30 days (Additional file 1: Fig. S16). Thus, we performed the hematological and histological analyses to compare the toxicity of the treatment on day 9. The serological examination showed severe cardiac, renal and hepatic toxicity in the DOX group, as confirmed by significant change in the hematological parameters, such as blood urea nitrogen (BUN), alanine transaminase (ALT) and troponin-I (Fig. 5b and Additional file 1: Fig. S17). In addition, mice treated with DOX also exhibited severe leukopenia, oligocythemia and thrombocytopenia in the complete blood count **(**CBC) analyses (Fig. 5c and Additional file 1: Fig. S18). In contrast, all the hematological parameters of F68-FDOX-treated mice were in normal range, which was similar with saline group, indicating greatly minimized DOX-related side effects. Finally, major organ tissues stained with H&E or TUNEL showed elevated structural abnormalities with apoptosis in DOX group, whereas F68-FDOX treatment did not induce noticeable tissue damages (Fig. 5d and Additional file 1: Fig. S19).

**Fig. 6:**
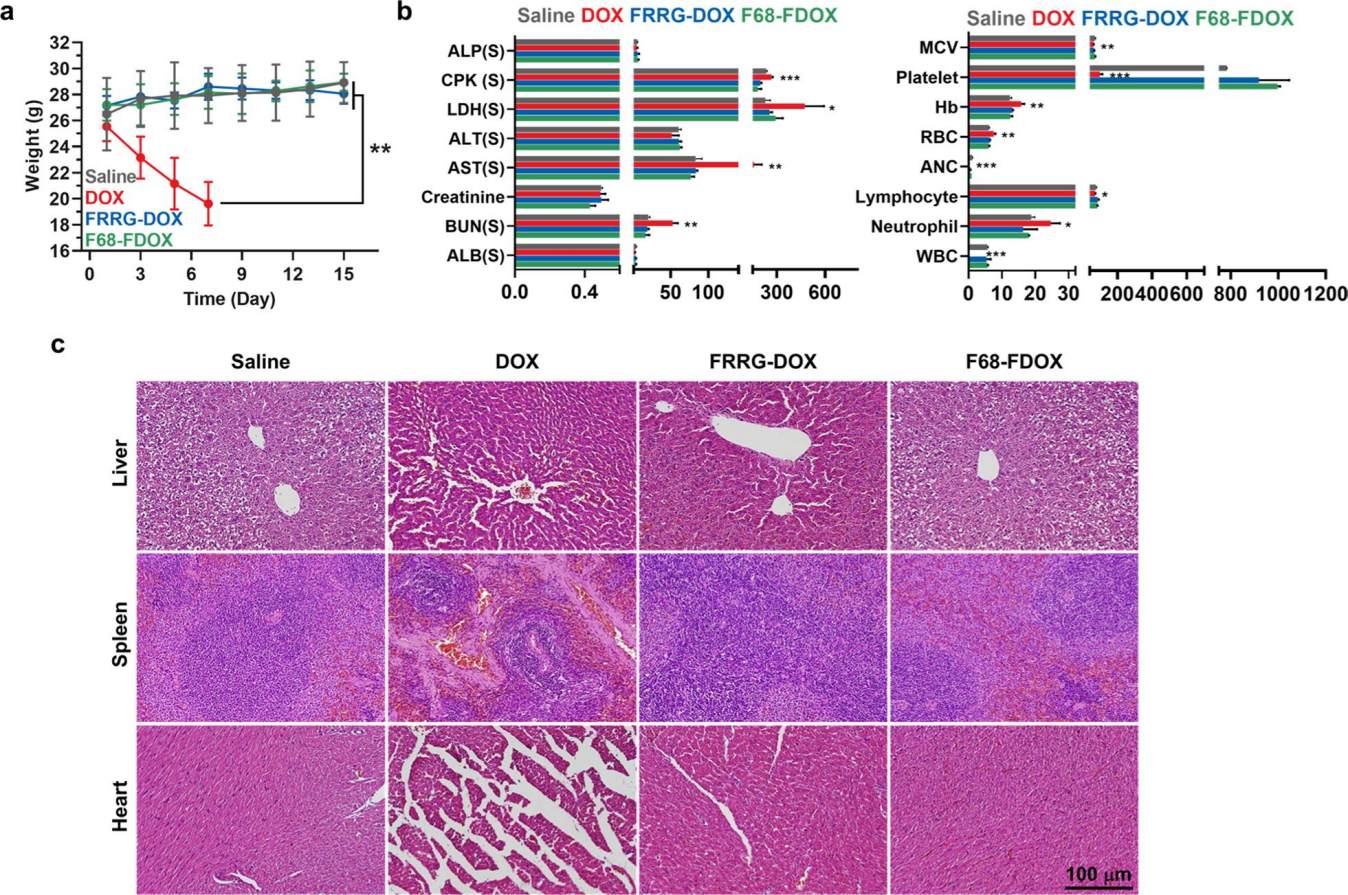
Toxicity study after multi-dosage. **a** Body weight change after multi-dosage with DOX, FRRG-DOX or F68-FDOX. **b** The hematological analyses on day 7 after multi-dosage with DOX, FRRG-DOX or F68-FDOX. **c** Major organ tissues stained with H&E on day 7 after multi-dosage with DOX, FRRG-DOX or F68-FDOX. Significance was determined by Tukey−Kramer *post-hoc* test

Next, we also assessed in vivo toxicity after each drug treatment five times with 3 days-intervals. As expected, systemic toxicity of DOX was more worsen owing to repetitive dose than in the single-dosage, showing severe body weight loss of the mice; accordingly, mice were all dead within 7 days of treatment (Fig. 6a and Additional file 1: Fig. S20). Even though same dose of equal drugs was administered into the mice, the outcomes can be varied according to the not only the number and intervals of the administration, but also different reactivity by species, feeding environment and age of the mice. Similar results by which the mice were all dead within two weeks after 5 mg/kg treatment of free DOX two times (Total 10 mg/kg) were reported, thus these observations are typical results when compared to other studies [42]. In contrast, F68-FDOX treatment showed high safety without significant body weight changes even with high doses of repeated injection. Hematological parameters that are confirmed on day 7 after DOX treatments remarkably got out from the normal range by severe organ dysfunction, while those of mice treated with F68-DOX were similar with saline group (Fig. 6b and Additional file 1: Fig. S21). Finally, histology of liver, spleen and heart tissues on day 7 showed severe tissue damages by DOX treatment, but F68-FDOX efficiently minimized the DOX-related systemic toxicity without damage to the normal organs (Fig. 6c). These results clearly demonstrate that F68-FDOX greatly minimize the DOX-related systemic toxicity accompanying severe cardiotoxicity by maintaining inactive state in normal tissues with innately low cathepsin B expression, improving safety of DOX-based chemotherapy.

## Conclusion

In this study, we reported the results about preclinical development of carrier-free doxorubicin prodrug nanoparticles (F68-FDOX) to enhance antitumor therapeutic potential with less toxicity. This new formulation has a potential to overcome several shortcomings of conventional nano-sized drug delivery system in the terms of technical- and industrial-aspects. With the precise and concise structure, they solved the most challenging problem of nanomedicines for clinical translation by allowing scale-up industrial production with easily controllable quality control (QC). The F68-FDOX induced a potent cytotoxicity preferentially in cancer cells by cathepsin B-specific cleavage mechanism, while maintained the inactive state in cathepsin B-deficient normal cells. In preclinical tumor models, F68-FDOX showed significantly improved PK/PD profiles and thereby passively accumulated in the tumor tissues via EPR effect. Importantly, F68-FDOX exhibited considerable antitumor activity with broad therapeutic spectrum in the multiple refractory tumor types, such as colon, breast and pancreatic cancers. Finally, their safety was clearly evaluated by confirming significantly minimized DOX-related systemic toxicity after single-dosage and even with high doses of repeated injection. Collectively, these results provide potential preclinical development process of an alternative approach, new formulation of carrier-free prodrug nanoparticles, for clinical translation of nanomedicines.

## Methods

### Reagents

N-terminal acylated Phe-Arg-Arg-Gly (FRRG) peptide was purchased from Peptron Co. (Daejeon, Republic of Korea). Dimethyl sulfoxide (DMSO), Dimethylformamide (DMF), doxorubicin hydrochloride (DOX), protease inhibitor cocktail, N,N-diisopropylethylamine (DIPEA), 1-ethyl-3-(3-dimethylaminopropyl)carbodiimide (EDC) and N-hydroxysuccinimide (NHS) were purchased from Sigma Aldrich (St. Louis, MO, USA). Cathepsin B, Cathepsin E, Cathepsin D, Cathepsin L, caspase-3 and TUNEL assay kit were purchased from R&D systems (Minneapolis, MN, USA). Cathepsin B siRNA and mono-clonal cathepsin B antibody were purchased from SantaCruz Biotechnology (Dallas,Texas, USA). Annexin V-Cy5 kit, RIPA buffer, streptavidin–horseradish peroxidase (streptavidin-HRP), BCA protein quantification kit, CellLight™ Early Endosomes-RFP, BacMam 2.0 and CellLight™ Lysosomes-RFP, BacMam 2.0 were purchased from Thermo Fisher Scientific Inc. (Rockford, IL, USA). Cell counting kit-8 (CCK-8) was purchased from Vitascientific (Beltsville, MD, USA). TEM grid (Carbon Film 200 Mesh copper) was purchased from Electron Microscopy Sciences (PA, USA). RPMI 1640 and DMEM media, antibiotics (streptomycin and penicillin) and fetal bovine serum (FBS) were purchased from WELGENE Inc. (Daegu, Republic of Korea). HT29 (human colon adenocarcinoma), MDA-MB231 (human breast adenocarcinoma), KPC960 (human pancreatic ductal adenocarcinoma) and H9C2 (rat BDIX heart myoblast) cell lines were purchased from American Type Culture Collection (ATCC; Manassas, VA, USA).

### Preparation and characterization of carrier-free prodrug nanoparticles

To prepare carrier-free prodrug nanoparticles, the cancer-specific prodrug was simply synthesized by conjugating cathepsin B-specific cleavable tetrapeptide (Phe-Arg-Arg-Gly; FRRG) to DOX via one-step reaction. Briefly, FRRG peptide (150 g, 1 eq), DOX (75.675 g, 0.7 eq), HATU (70.875 g, 1 eq) and DIPEA (2 eq) were dissolved in DMF (2 L), followed by stirring at 10 °C for 3 h. The FRRG-DOX was purified using Sep-Pak C18 column chromatography and the resulting filtrate was analyzed via RP-HPLC (Agilent 1200 Series HPLC System). Finally, the purified FRRG-DOX was lyophilized for 3 days to obtain as a red powder (111.75 g, yield: 78%). The molecular weight and chemical structure of FRRG-DOX were characterized by matrix-assisted laser desorption/ionization time of flight mass spectrometer (MALDI-TOF, cyano-4-hydroycinnamic acid (CHCA) matrix, AB Sciex TOF/TOF 5800 System, USA) and ^1^H-NMR (DD2 FT NMR, Agilent Technologies, USA), respectively. The red powder of FRRG-DOX was dispersed in aqueous condition for self-assembly of prodrugs. To stabilize the FRRG-DOX nanoparticles with Pluronic F68, Pluronic F68 solution (30% v/v) was slowly added into FRRG-DOX solution in the distilled water condition, followed by lyophilization for 3 days, resulting in carrier-free prodrug nanoparticles (F68-FDOX). The size distribution and zeta potential of FRRG-DOX and F68-FDOX nanoparticles (1 mg/mL in saline) were analyzed using a Zetasizer Nano ZS (Malvern Instruments, Worcestershire, UK), and their particle morphology was characterized in distilled water (1 mg/mL) using a transmission electron microscopy (TEM, CM-200, Philips, USA). The long-term storage stability of FRRG-DOX power was assessed after storage of 3, 6, 12 months in the low (− 4 °C), room (37 °C) or accelerated (60 °C) condition, followed by analysis of size, chemical structure and purity as described above. The cathepsin B-specific cleavage of F68-FDOX was assessed after incubation with various enzymes. Briefly, F68-FDOX was incubated with MES buffer containing 10 μg of cathepsin B enzyme at 37 °C and were analyzed using RP-HPLC. As control, the F68-FDOX was also incubated with 10 μg of different enzymes (Cathepsin D, E, L and Caspase-3) for 24 h.

### Cellular uptake of F68-FDOX

The cellular uptake of F68-FDOX was assessed in the HT29, MDA-MB231, KPC960 and H9C2 cell lines. Briefly, 1 × 10^5^ of each cell was seeded into glass-bottom confocal dishes, followed by incubation with F68-FDOX or DOX (1 μM) for 48 h at 37 °C. To monitor intracellular localization of F68-FDOX, the endosomes and lysosomes in the HT29 cells were labeled with Rab5a-RFP or Lamp1-RFP fusion constructs (1 μM) for 1 h at 37 °C, respectively. Then, cells were washed with DPBS three times, fixed with paraformaldehyde fixative for 15 min, and stained with DAPI solution for 10 min in the dark (Invitrogen, Carlsbad, CA). Finally, the cells were observed using a confocal laser scanning microscope (CLSM) equipped with 405 diode (405 nm) and HeNe-Red (633 nm) lasers (Leica, Germany). Co-localization of the F68-FDOX and Rab5a-RFP (endosomes) or Lamp1-RFP (lysosomes) was analyzed using an Image-Pro software (Media Cybernetic, Rockville, MD, USA).

### Cytotoxicity study

The cytotoxicity of F68-FDOX was assessed via a cell counting kit-8 (CCK-8) assays. First, 1 × 10^5^ HT29, MDA-MB231, KPC960 or H9C2 cells were seeded into 96-well cell culture plates. After 24 h of stabilization, the F68-FDOX or free DOX were treated to each cell for 48 h, followed by additional incubation with with culture medium containing CCK-8 solution (10%) for 20 min. Finally, cell viability was measured by a microplate reader (VERSAmaxTM; Molecular Devices Corp., USA) with 450 nm of wavelength.

### Biodistribution of F68-FDOX

The 6-week male BALB/c and BALB/c nu/nu mice were purchased from NaraBio (Gyeonggi-do, Republic of Korea). Mice were bred under pathogen-free conditions in the Korea Institute of Science and Technology (KIST). All experiments with animals were performed in compliance with the relevant laws and institutional guidelines of Institutional Animal Care and Use Committee (IACUC; approved number of 2020-123) in Korea Institute of Science and Technology (KIST). First, pharmacokinetics (PK) profiles were assessed in the BALB/c mice after intravenous injection with DOX (4 mg/kg), FRRG-DOX (4 mg/kg based on DOX contents) or F68-FDOX (4 mg/kg based on DOX contents). After treatment, blood samples were collected from mice by cardiac puncture after deep anesthesia at pre-determined times, followed by analysis with HPLC with fluorescence detector. The PK parameters including area under the curves (AUC), clearance (CL), volume of distribution (Vd) and half-life (*t*_1/2_) were calculated using a WinNonlin software. The tumor targeting of PD-NPs was assessed in HT29 tumor-bearing mice, which were prepared by subcutaneous inoculation of 1 × 10^7^ HT29 cells. The NIRF imaging was performed after 9 h of injection of DOX (4 mg/kg), FRRG-DOX (4 mg/kg based on DOX contents) or F68-FDOX (4 mg/kg based on DOX contents). The fluorescence intensities in the tumor regions were quantified using a Living Image software (PerkinElmer, Waltham, MA, USA). The ex vivo NIRF imaging of collected major organs after 9 h of injection was also performed using IVIS Lumina Series III system. The pharmacodynamics (PD) of F68-FDOX was assessed by histological analysis of major organ (liver, lung, spleen, kidney and heart) and tumor tissues of mice after 9 h of injection with DOX, FRRG-DOX or F68-FDOX. For this analysis, each tissue was cut into 8-μm sections using rotary microtome and analyzed via confocal laser scanning microscope (CLSM) equipped with 405 diode (405 nm) and HeNe-Red (633 nm) lasers.

### Antitumor activity of F68-FDOX in colon, breast and pancreatic cancer models

The antitumor activity was evaluated in colon, breast and pancreatic cancer models, which were prepared by subcutaneous injection with 1 × 10^7^ HT29, MDA-MB231 or KPC960 cells, respectively. When the tumor volumes were approximately 80 mm^3^, mice were randomly divided into four groups: (i) saline; (ii) DOX (4 mg/kg); (iii) FRRG-DOX (4 mg/kg based on DOX contents); and (iv) F68-FDOX (4 mg/kg based on DOX contents). The mice were treated once every three days, and tumor volumes were calculated as the largest diameter × smallest diameter^2^ × 0.53, every 2 days. The mice with a tumor size of 2000 mm^3^ or higher were counted as dead. To analyze the antitumor activity in a single cell level, the tumor tissues were collected on day 9, and single cell were isolated from the tumor tissues using a Tumor Dissociation Kit. After cell counting, single cells were stained with Annexin V for 1 h in room temperature and analyzed via flow cytometer.

### Toxicity study of F68-FDOX treatment

The safety of F68-FDOX treatment was assessed by histological and hematological analyses. Briefly, DOX (4 mg/kg), FRRG-DOX (4 mg/kg based on DOX contents) or F68-FDOX (4 mg/kg based on DOX contents) were intravenously injected into BALB/c mice. On day 9 after treatments, major organs were collected from mice, and structural abnormalities and apoptosis in organ tissues were assessed by staining with H&E or TUNEL, respectively. In case of hematological analyses, blood samples were collected from mice on day 9. For the complete blood count (CBC) analyses, each blood sample was mixed with EDTA, and a portion of blood sample was centrifuged at 2100 rpm for 20 min to obtain blood plasma. The following factors in blood samples were measured; albumin/globulin ratio (A/G), troponin-I, albumin (Alb), total protein (TP), alanine aminotransferase (ALT), total cholesterol (T-Chol), blood urea nitrogen (BUN), alkaline phosphatase (ALP), red blood cell (RBC), white blood cell (WBC), mean corpuscular hemoglobin (MCH), mean corpuscular volume (MCV), hematocrit (HCT), hemoglobin (HGB) and platelet (PLT). To assess the safety of F68-FDOX treatment after multiple-dosage, DOX (4 mg/kg), FRRG-DOX (4 mg/kg based on DOX contents) or F68-FDOX (4 mg/kg based on DOX contents) were injected into mice once every three days. Then, histological and hematological analyses were performed as described above.

### Statistics

The statistical significance between two groups was analyzed using Student’s t-test. One-way analysis of variance (ANOVA) was performed for comparisons of more than two groups, and multiple comparisons were analyzed using the Tukey–Kramer post hoc test. Survival data was plotted as Kaplan–Meier curves and analyzed using the log-rank test. The statistical significance was indicated with asterisks (*p < 0.05, **p < 0.01, ***p < 0.001) in the figures.

## Supplementary Information

**Additional file 1: Figure S1.** Synthetic route to prepare the cancer-specific prodrug FRRG-DOX. **Figure S2.** The **(a)** purity, **(b)** exact mass and **(c)** chemical structure of FRRG-DOX, as confirmed via HPLC, MALDI-TOF and ^1^H-NMR, respectively. **Figure S3.** Detail information of the particle stability analysis of **(a)** FRRG-DOX and **(b)** F68-FDOX nanoparticles in mouse serum. **Figure S4.** Cumulative release of G-DOX from F68-FDOX after incubation with cathepsin B. **Figure S5.** Cleavage behavior of FRRG-DOX after incubation with cathepsin B. **Figure S6.** The mass analysis of the newly appeared peak (13 min; Fig. 1f) in the HPLC spectrum after incubation of F68-FDOX with cathepsin B. **Figure S7.** Long-term storage stability of lyophilized F68-FDOX powder stored for **(a)** 3, **(b)** 6, **(c)** 12 months in the low (-4 °C) condition. **Figure S8.** Long-term storage stability of lyophilized F68-FDOX powder stored for **(a)** 3, **(b)** 6, **(c)** 12 months in the room (37 °C) condition. **Figure S9.** Long-term storage stability of lyophilized F68-FDOX powder stored for **(a)** 3, **(b)** 6, **(c)** 12 months in the accelerated (60 °C) condition. **Figure S10.** The cellular uptake of F68-FDOX and DOX in the HT29, MDA-MB231, KPC960 and H9C2 cells after 6 or 24 h of incubation. **Figure S11.** The fluorescence intensity profile was measured from the line-scans through cells of white lines in the fluorescence imaging results in Fig. 2b. **Figure S12.** The mass analysis of the DOX released from F68-FDOX in the HT29, MDA-MB231 and KPC960 cells after 48 h of treatment. **Figure S13.** The cell viability of HT29, MDA-MB231, KPC960 and H9C2 cells after 48 h treatment with FRRG-DOX. **Figure S14.** Quantitative analysis for the fluorescence intensity in major organs and tumor tissues of HT29-tumor bearing mice after 9 h of treatment with DOX, FRRG-DOX or F68-FDOX. **Figure S15.** Quantitative analysis for the apoptosis region of tumor tissues stained with TUNEL. **Figure S16.** Mice survival after single-dosage with DOX, FRRG-DOX or F68-FDOX. **Figure S17.** Detail information of the serological examination on day 9 after single-dosage with DOX, FRRG-DOX or F68-FDOX. **Figure S18.** Detail information of the complete blood count **(**CBC) analyses on day 9 after single-dosage with DOX, FRRG-DOX or F68-FDOX. **Figure S19.** Major organ tissues stained with TUNEL on day 9 after single-dosage with DOX, FRRG-DOX or F68-FDOX. **Figure S20.** Mice survival after multi-dosage with DOX, FRRG-DOX or F68-FDOX. **Figure S21.** Detail information of the hematological analyses on day 9 after multi-dosage with DOX, FRRG-DOX or F68-FDOX.

## Data Availability

All relevant data are available with the article and its additional files, or available the corresponding authors upon reasonable requests.
