# Supplementary material for: Preclinical development of carrier-free prodrug nanoparticles for enhanced antitumor therapeutic potential with less toxicity
Source: J Nanobiotechnology. 2022 Oct 4;20:436. doi: 10.1186/s12951-022-01644-x (PMC9531438; doi:10.1186/s12951-022-01644-x)
Supplement: Supplementary file 1 — Additional file 1: Figure S1. Synthetic route to prepare the cancer-specific prodrug FRRG-DOX. Figure S2. The (a) purity, (b) exact mass and (c) chemical structure of FRRG-DOX, as confirmed via HPLC, MALDI-TOF and 1H-NMR, respectively. Figure S3. Detail information of the particle stability analysis of (a) FRRG-DOX and (b) F68-FDOX nanoparticles in mouse serum. Figure S4. Cumulative release of G-DOX from F68-FDOX after incubation with cathepsin B. Figure S5. Cleavage behavior of FRRG-DOX after incubation with cathepsin B. Figure S6. The mass analysis of the newly appeared peak (13 min; Fig. 1f) in the HPLC spectrum after incubation of F68-FDOX with cathepsin B. Figure S7. Long-term storage stability of lyophilized F68-FDOX powder stored for (a) 3, (b) 6, (c) 12 months in the low (-4 °C) condition. Figure S8. Long-term storage stability of lyophilized F68-FDOX powder stored for (a) 3, (b) 6, (c) 12 months in the room (37 °C) condition. Figure S9. Long-term storage stability of lyophilized F68-FDOX powder stored for (a) 3, (b) 6, (c) 12 months in the accelerated (60 °C) condition. Figure S10. The cellular uptake of F68-FDOX and DOX in the HT29, MDA-MB231, KPC960 and H9C2 cells after 6 or 24 h of incubation. Figure S11. The fluorescence intensity profile was measured from the line-scans through cells of white lines in the fluorescence imaging results in Fig. 2b. Figure S12. The mass analysis of the DOX released from F68-FDOX in the HT29, MDA-MB231 and KPC960 cells after 48 h of treatment. Figure S13. The cell viability of HT29, MDA-MB231, KPC960 and H9C2 cells after 48 h treatment with FRRG-DOX. Figure S14. Quantitative analysis for the fluorescence intensity in major organs and tumor tissues of HT29-tumor bearing mice after 9 h of treatment with DOX, FRRG-DOX or F68-FDOX. Figure S15. Quantitative analysis for the apoptosis region of tumor tissues stained with TUNEL. Figure S16. Mice survival after single-dosage with DOX, FRRG-DOX or F68-FDOX. Figure S17. Detail inform [file 12951_2022_1644_MOESM1_ESM.docx]

**Supplementary Information for**

Preclinical development of carrier-free prodrug nanoparticles for enhanced antitumor therapeutic potential with less toxicity

Man Kyu Shim^1,#^, Suah Yang^1,#^, Jooho Park^1^, Jun Sik Yoon^1^, Jinseong Kim^1,2^, Yujeong Moon^1,3^, Nayeon Shim^1,4^, Mihee Jo^1^, Yongwhan Choi^1^, Kwangmeyung Kim^1,4,*^

^1^Center for Theragnosis, Biomedical Research Institute, Korea Institute of Science and Technology, Seoul, 02792, Republic of Korea.

^2^KU-KIST Graduate School of Converging Science and Technology, Korea University, Seoul, 02841, Republic of Korea.

^3^Department of Bioengineering, Korea University, Seoul, 02841, Republic of Korea.

^4^College of Pharmacy, Graduate School of Pharmaceutical Sciences, Ewha Womans University, Seoul 03760, Republic of Korea.

^#^These authors contributed equally to this work.

*Correspondence and requests for materials should be addressed to **K. Kim** (E-mail: kimkm@ewha.ac.kr; address: College of Pharmacy, Graduate School of Pharmaceutical Sciences, Ewha Womans University, Seoul 03760, Republic of Korea).

**
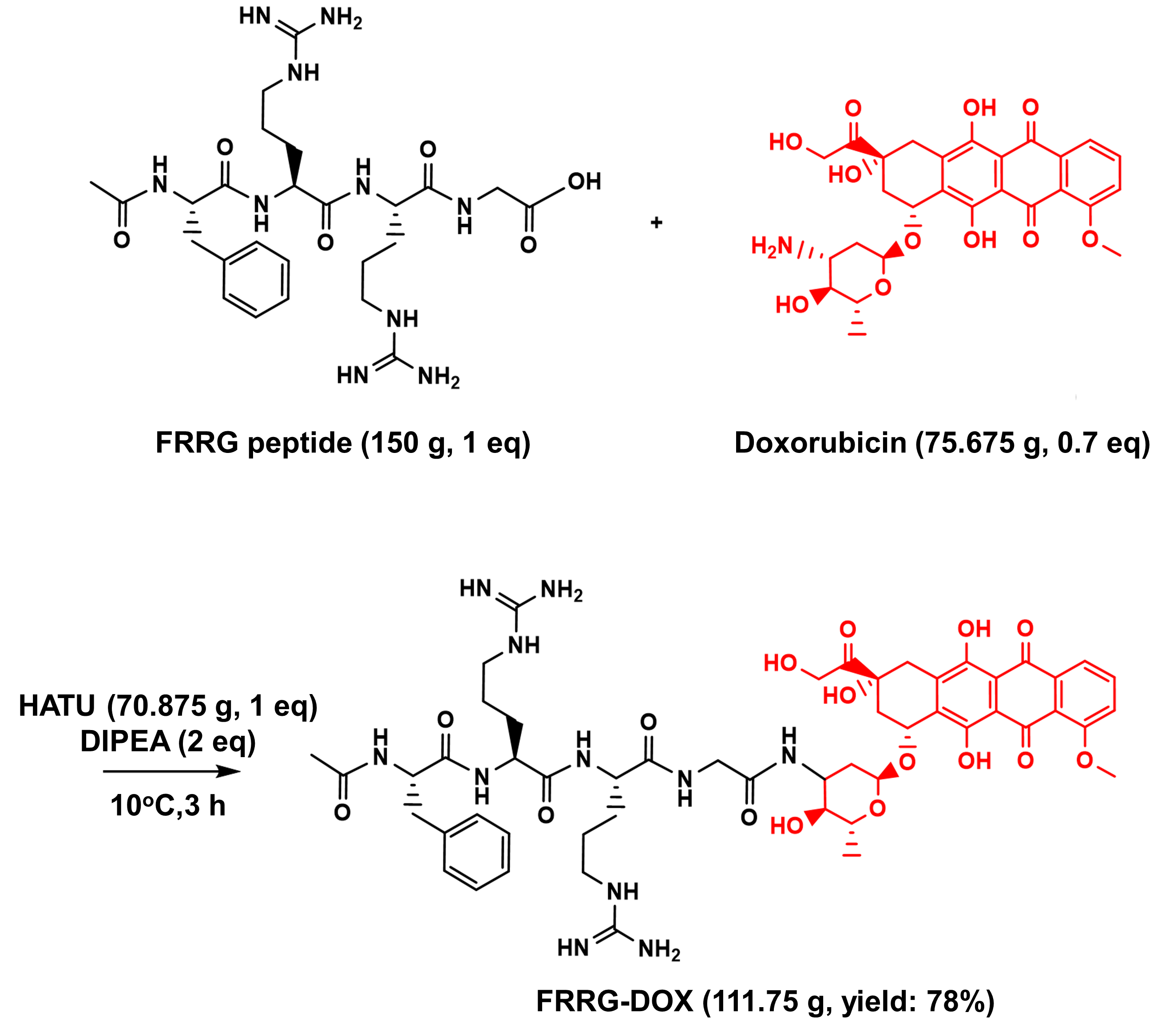
**

**Figure S1.** Synthetic route to prepare the cancer-specific prodrug FRRG-DOX.


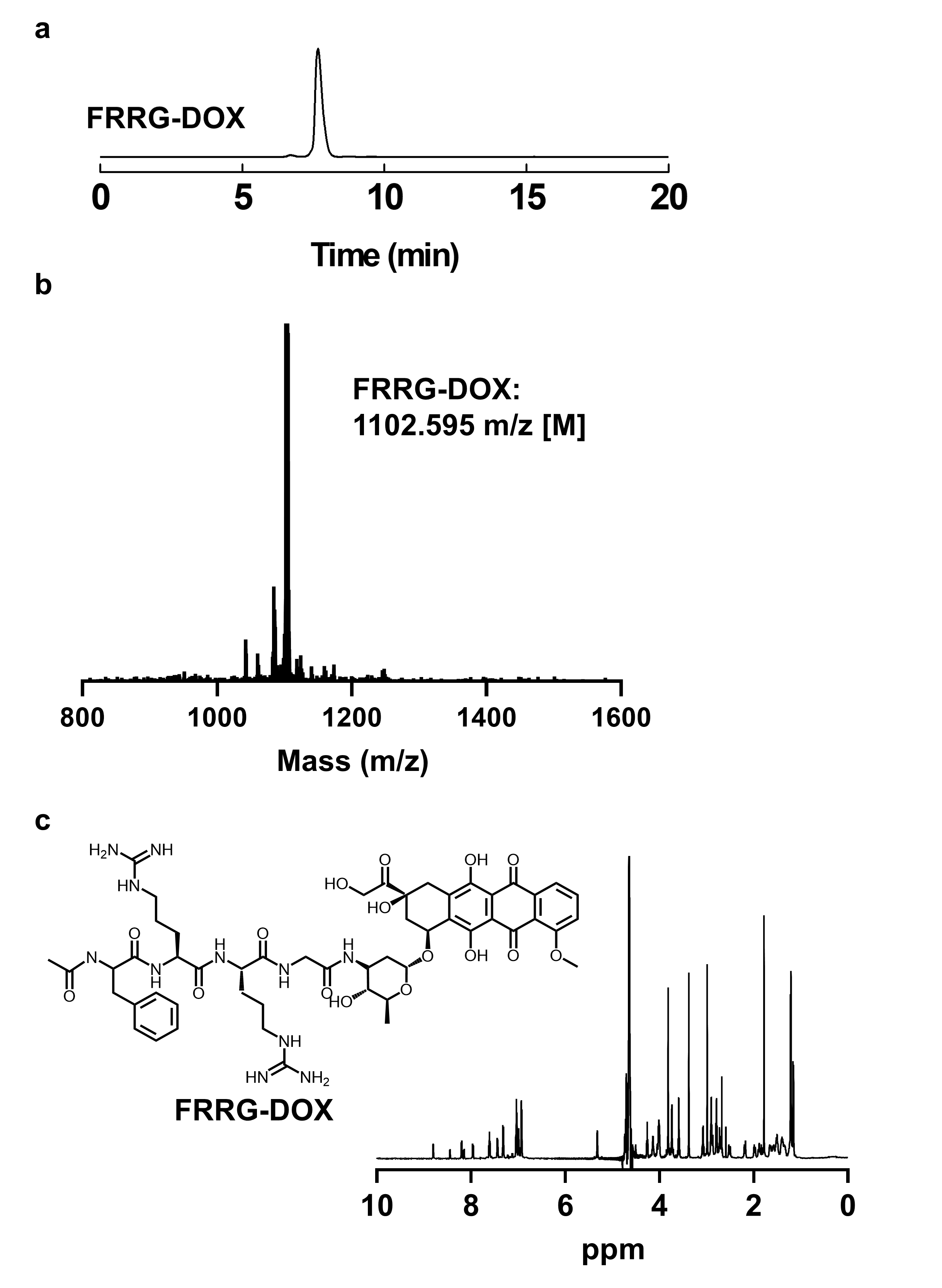


**Figure S2.** The **(a)** purity, **(b)** exact mass and **(c)** chemical structure of FRRG-DOX, as confirmed *via* HPLC, MALDI-TOF and ^1^H-NMR, respectively.


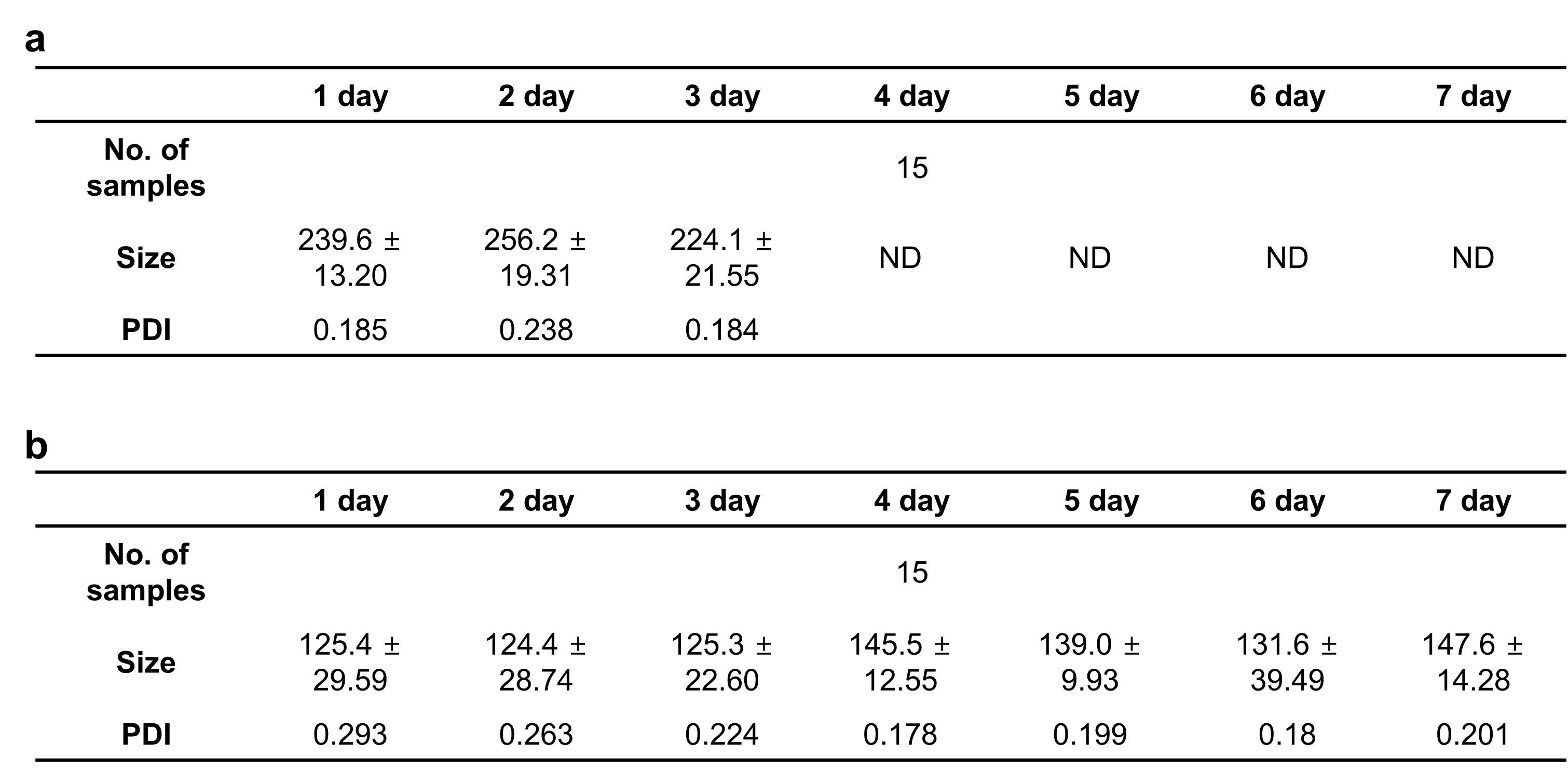


**Figure S3.** Detail information of the particle stability analysis of **(a)** FRRG-DOX and **(b)** F68-FDOX nanoparticles in mouse serum.

**Figure S4.** Cumulative release of G-DOX from F68-FDOX after incubation with cathepsin B.


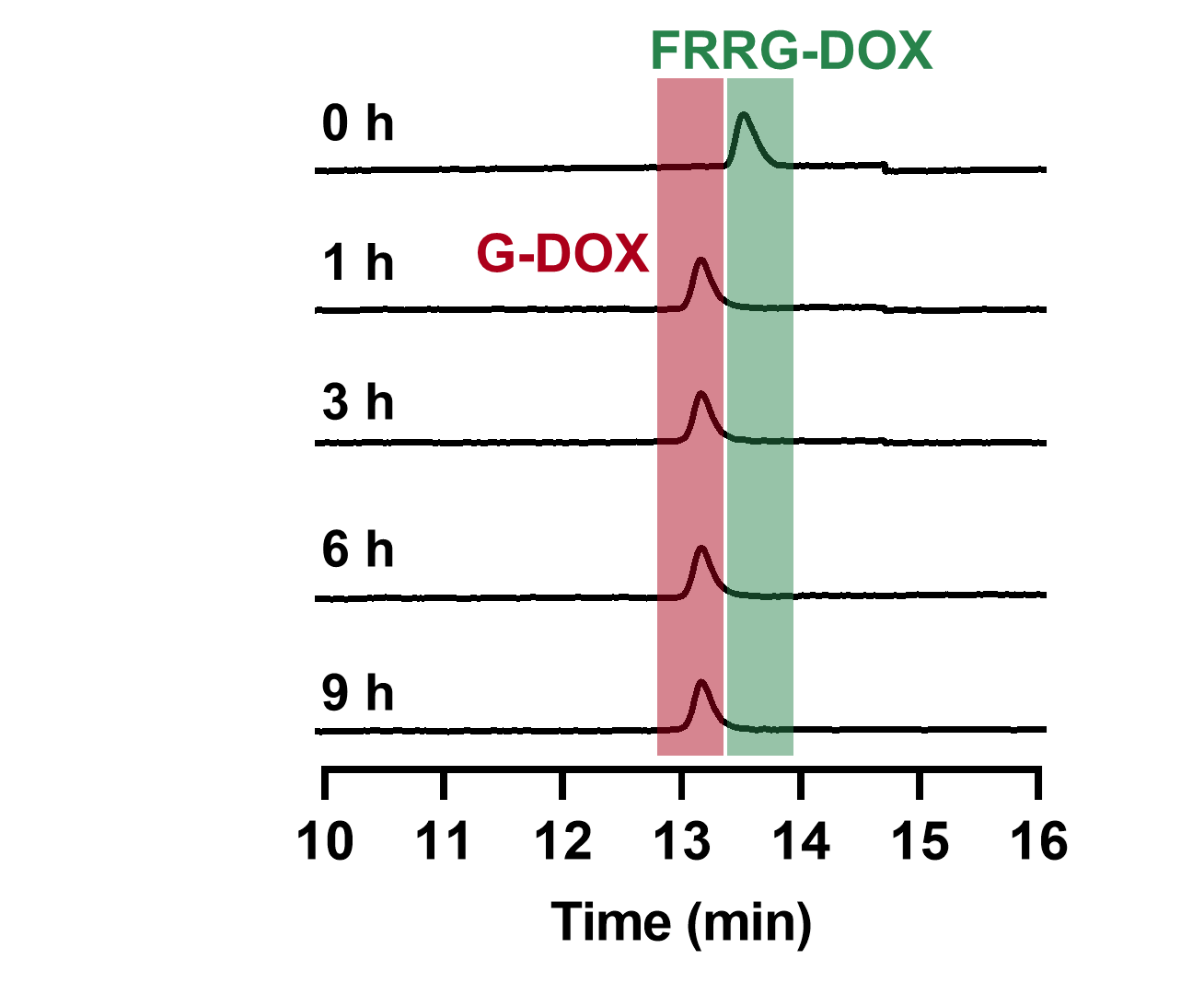


**Figure S5.** Cleavage behavior of FRRG-DOX after incubation with cathepsin B.


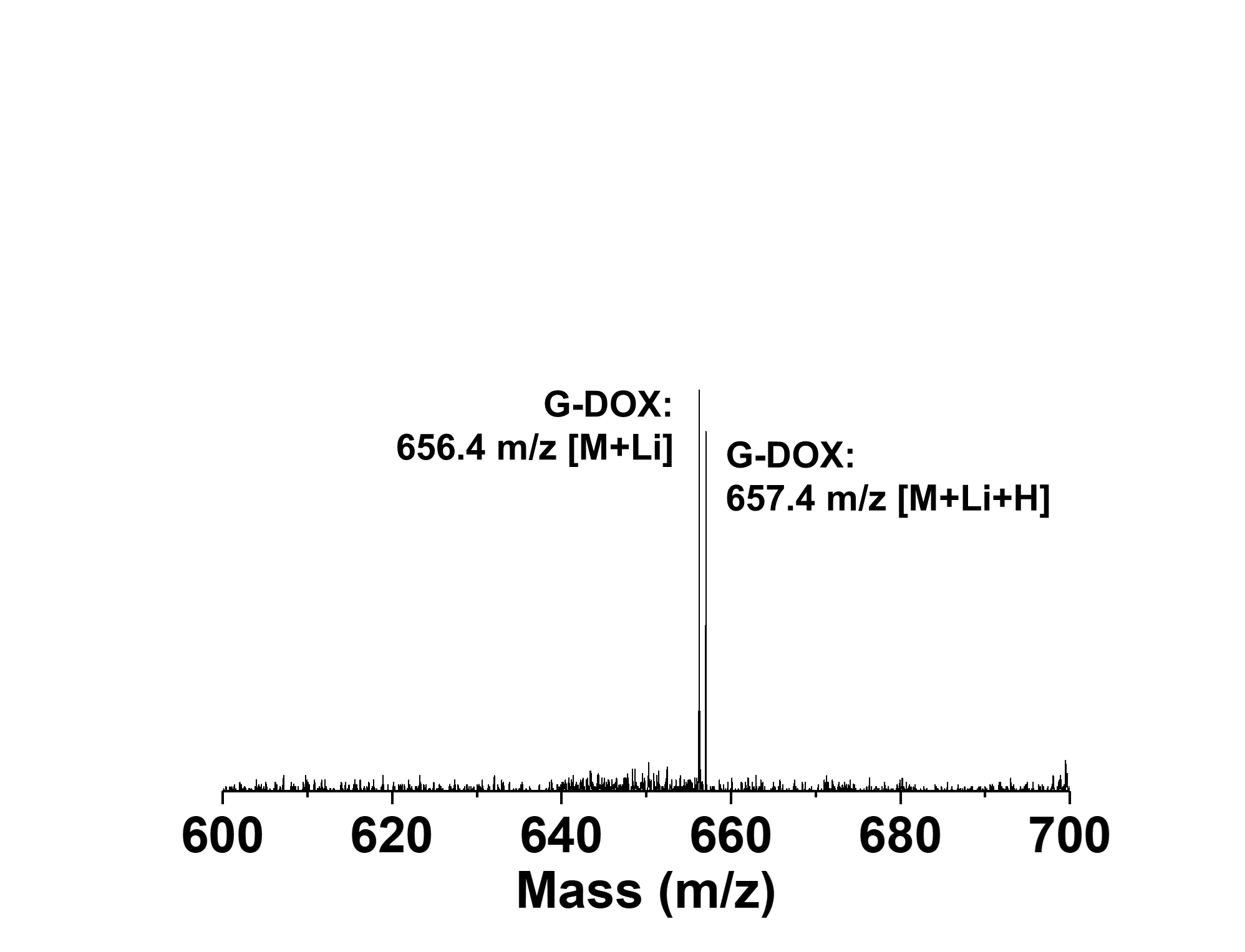


**Figure S6.** The mass analysis of the newly appeared peak (13 min; Figure 1f) in the HPLC spectrum after incubation of F68-FDOX with cathepsin B.


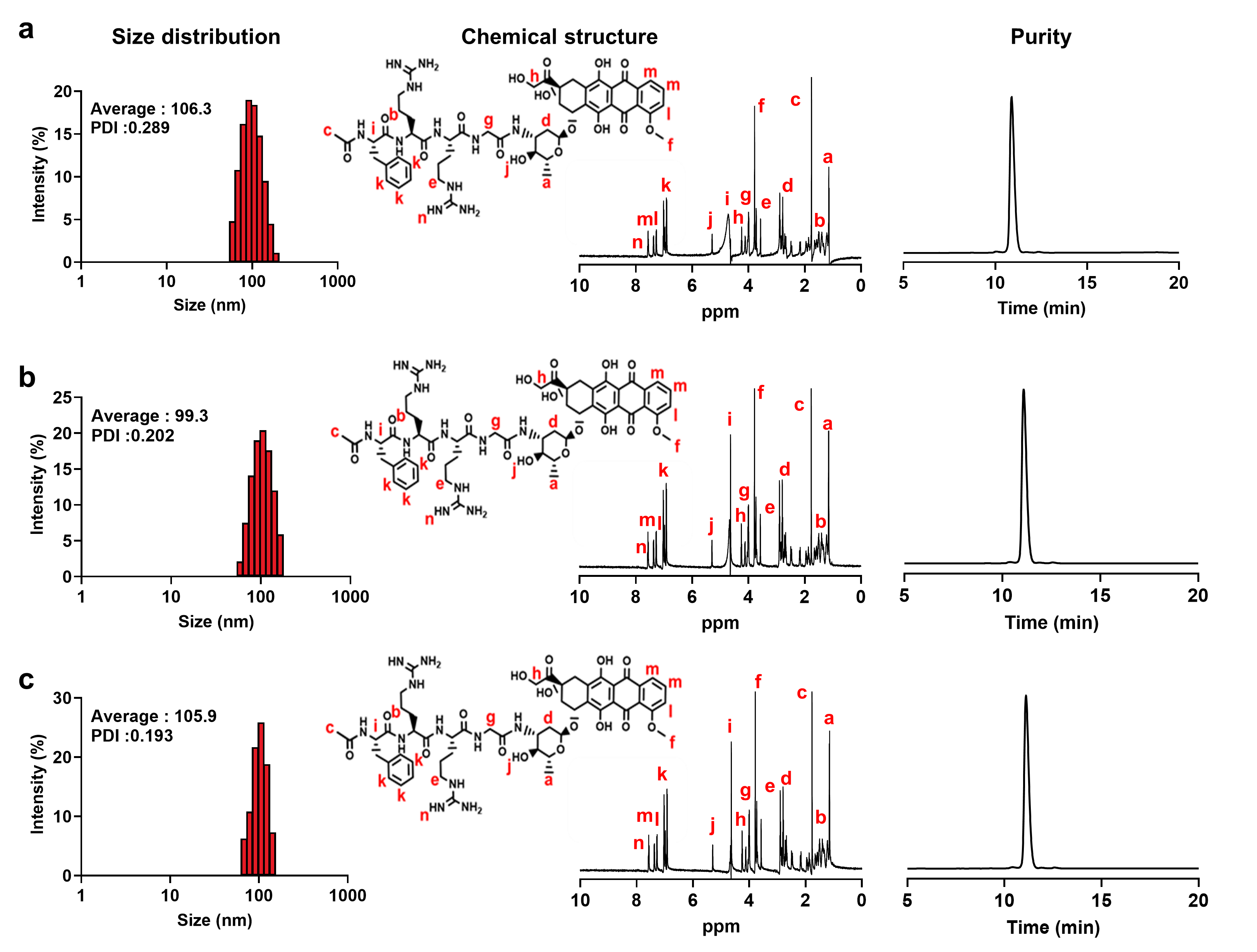


**Figure S7.** Long-term storage stability of lyophilized F68-FDOX powder stored for **(a)** 3, **(b)** 6, **(c)** 12 months in the low (-4^o^C) condition.


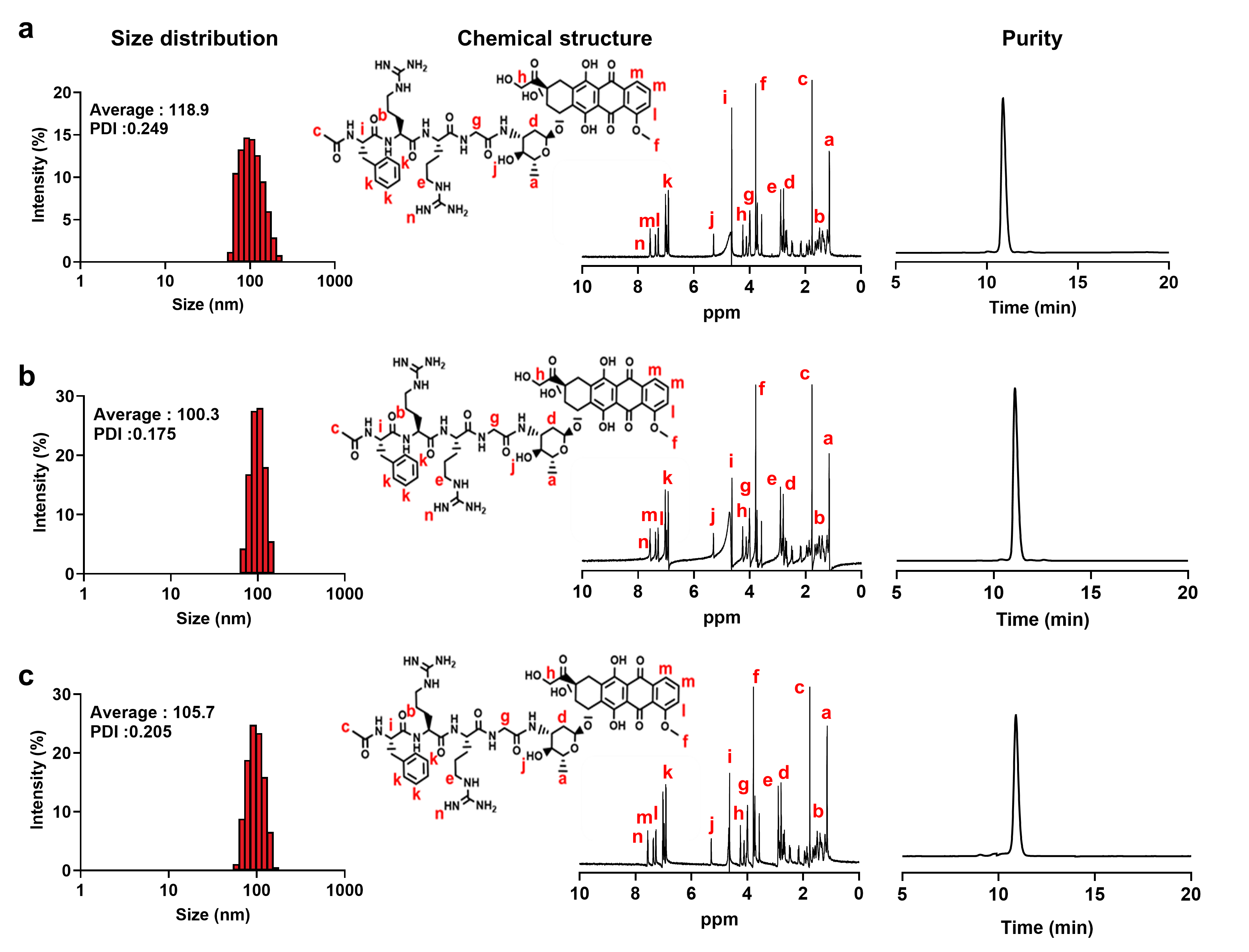


**Figure S8.** Long-term storage stability of lyophilized F68-FDOX powder stored for **(a)** 3, **(b)** 6, **(c)** 12 months in the room (37^o^C) condition.

**
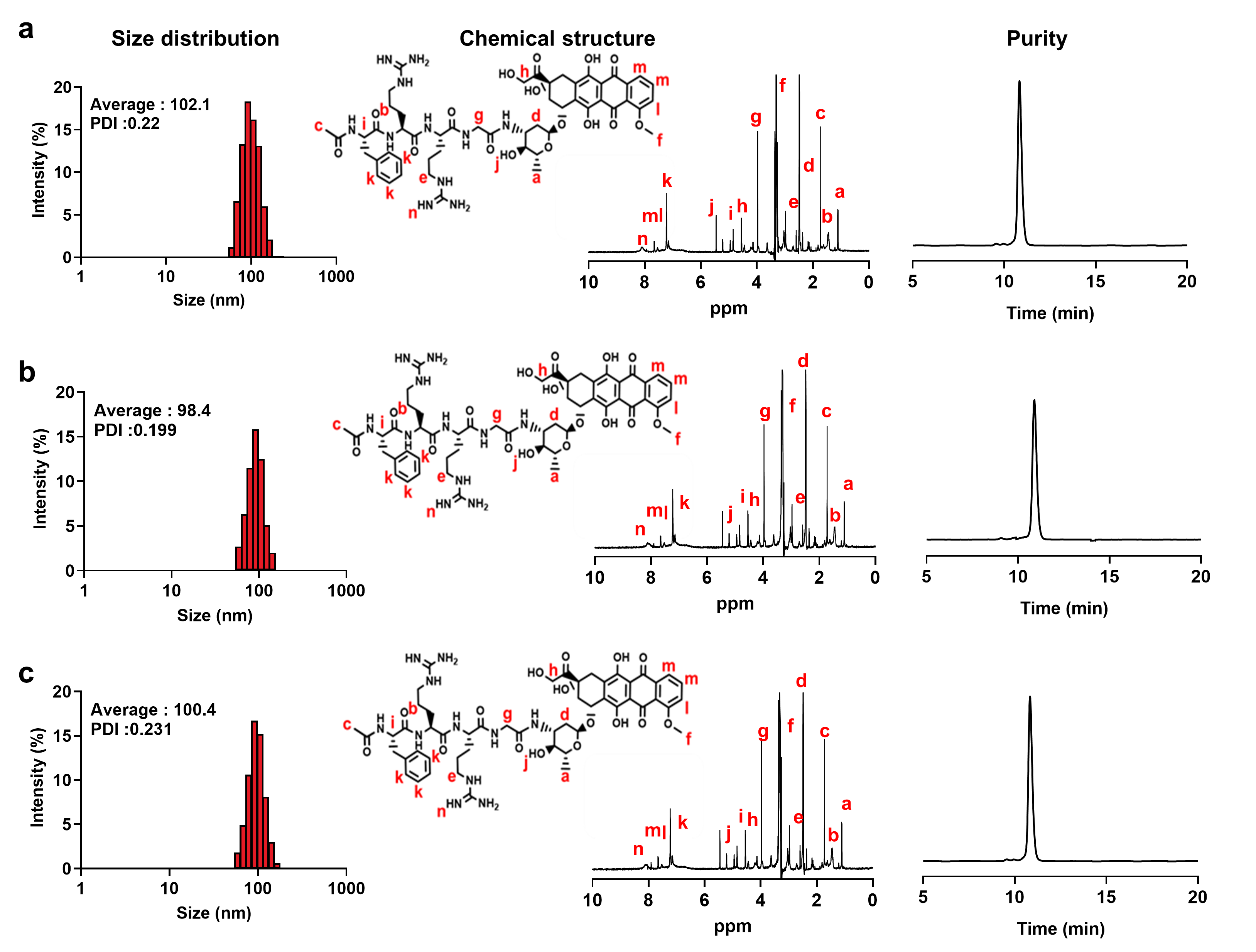
**

**Figure S9.** Long-term storage stability of lyophilized F68-FDOX powder stored for **(a)** 3, **(b)** 6, **(c)** 12 months in the accelerated (60^o^C) condition.


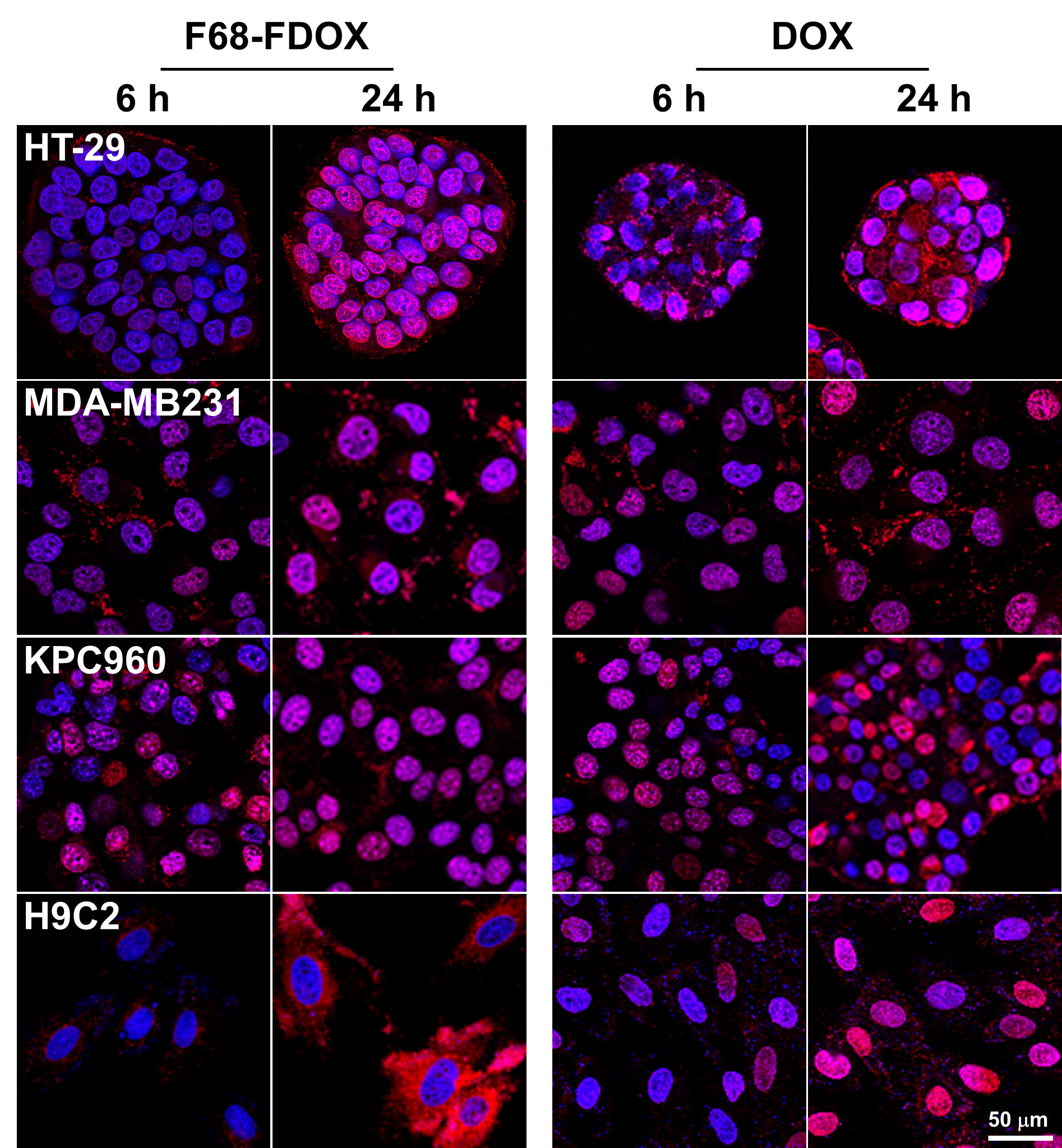


**Figure S10.** The cellular uptake of F68-FDOX and DOX in the HT29, MDA-MB231, KPC960 and H9C2 cells after 6 or 24 h of incubation.


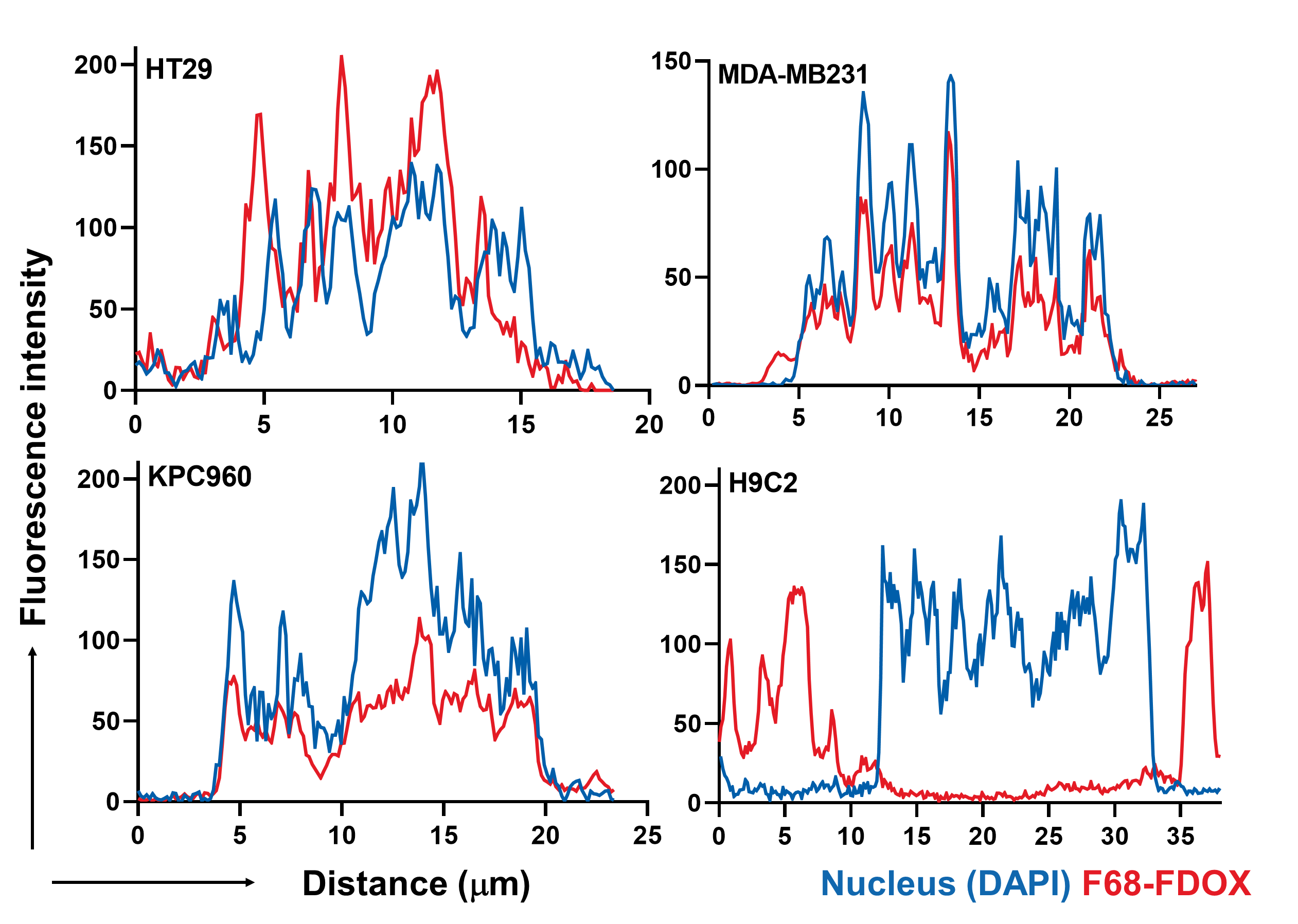


**Figure S11.** The fluorescence intensity profile was measured from the line-scans through cells of white lines in the fluorescence imaging results in Figure 2b.


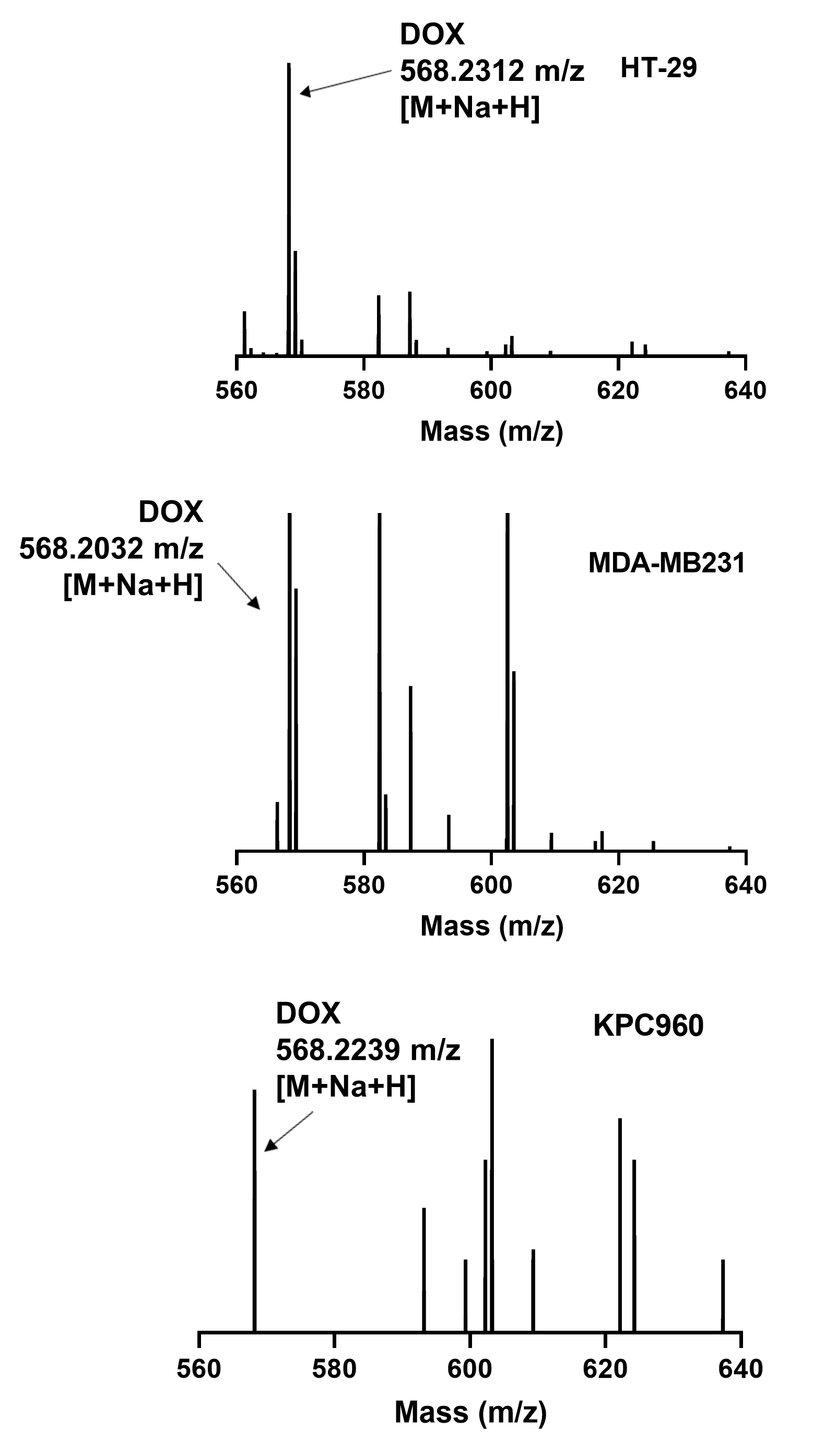


**Figure S12.** The mass analysis of the DOX released from F68-FDOX in the HT29, MDA-MB231 and KPC960 cells after 48 h of treatment.

**Figure S13.** The cell viability of HT29, MDA-MB231, KPC960 and H9C2 cells after 48 h treatment with FRRG-DOX.

**Figure S14.** Quantitative analysis for the fluorescence intensity in major organs and tumor tissues of HT29-tumor bearing mice after 9 h of treatment with DOX, FRRG-DOX or F68-FDOX.

**Figure S15.** Quantitative analysis for the apoptosis region of tumor tissues stained with TUNEL.

**Figure S16.** Mice survival after single-dosage with DOX, FRRG-DOX or F68-FDOX.

**
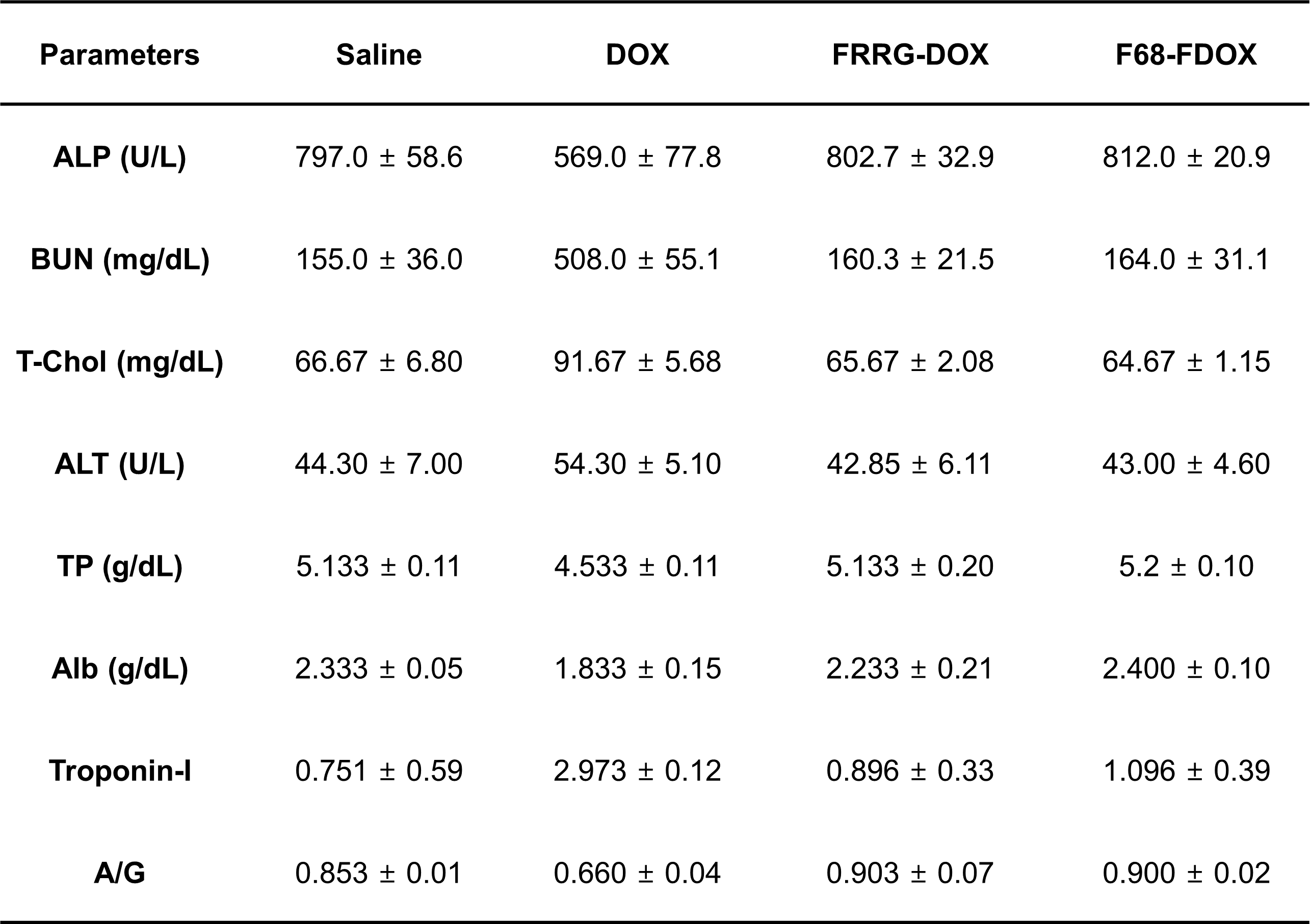
**

**Figure S17.** Detail information of the serological examination on day 9 after single-dosage with DOX, FRRG-DOX or F68-FDOX.


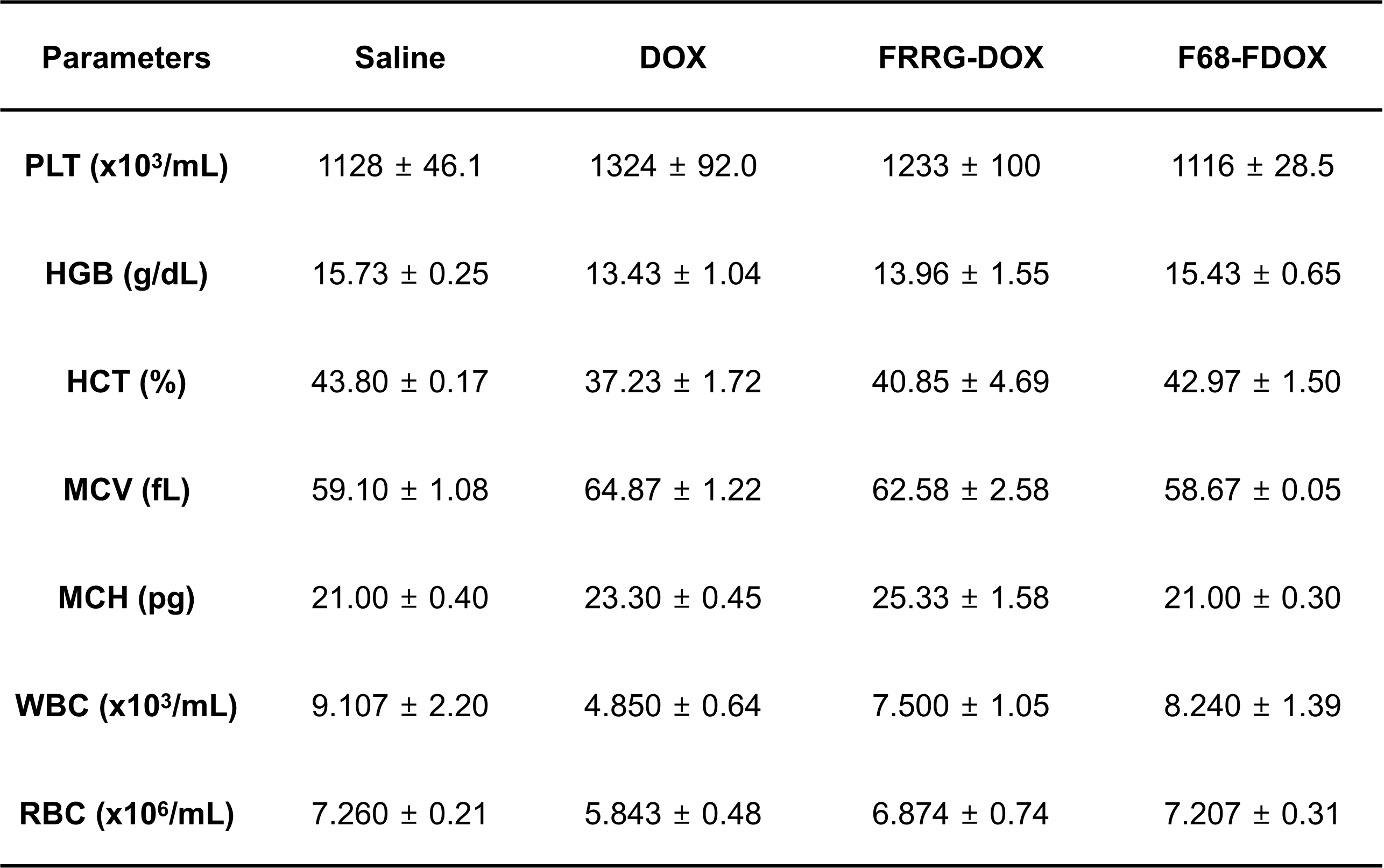


**Figure S18.** Detail information of the complete blood count **(**CBC) analyses on day 9 after single-dosage with DOX, FRRG-DOX or F68-FDOX.


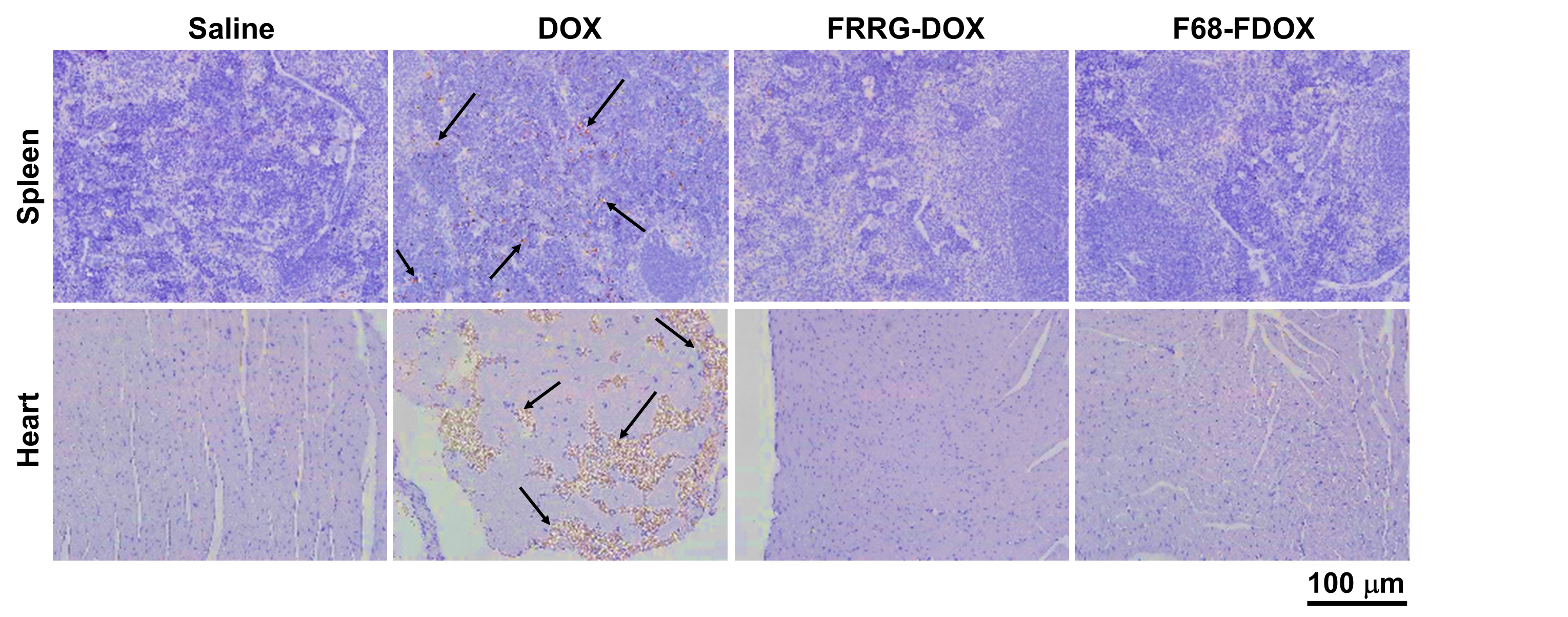


**Figure S19.** Major organ tissues stained with TUNEL on day 9 after single-dosage with DOX, FRRG-DOX or F68-FDOX.

**Figure S20.** Mice survival after multi-dosage with DOX, FRRG-DOX or F68-FDOX.


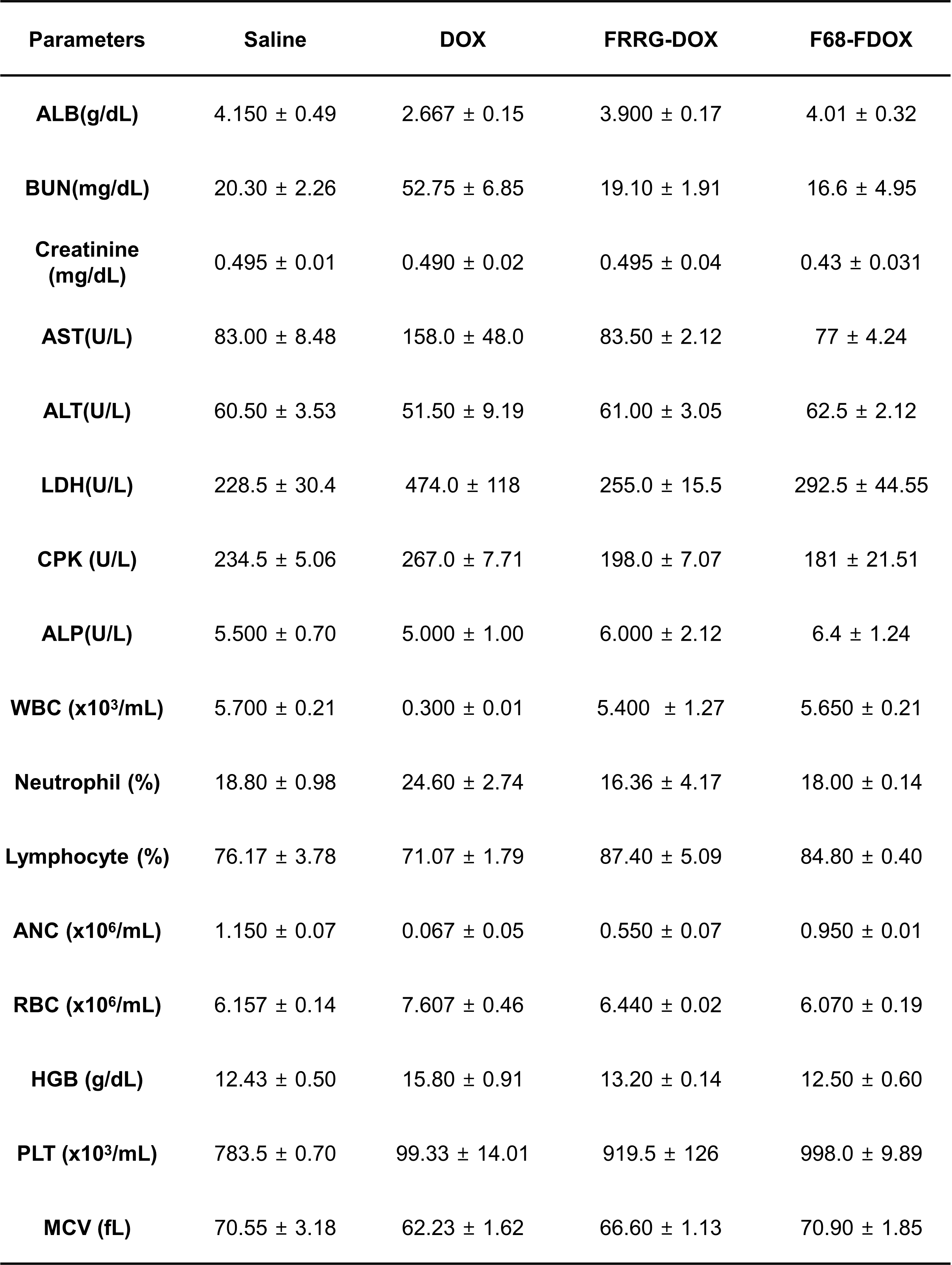


**Figure S21.** Detail information of the hematological analyses on day 7 after multi-dosage with DOX, FRRG-DOX or F68-FDOX.
